# Usnic acid modifies MRSA drug resistance through down‐regulation of proteins involved in peptidoglycan and fatty acid biosynthesis

**DOI:** 10.1002/2211-5463.12650

**Published:** 2019-09-30

**Authors:** Sneha Sinha, Vivek Kumar Gupta, Parmanand Kumar, Rajiv Kumar, Robin Joshi, Anirban Pal, Mahendra P. Darokar

**Affiliations:** ^1^ Molecular Bioprospection Department CSIR‐Central Institute of Medicinal and Aromatic Plants Lucknow India; ^2^ Biotechnology Division CSIR‐Institute of Himalayan Bioresource Technology Palampur India; ^3^ Present address: Department of Biotechnology Indian Institute of Technology Roorkee‐247667 India

**Keywords:** drug resistance reversal, MRSA, phytomolecules, *Staphylococcus aureus*, synergy, usnic acid

## Abstract

Multidrug‐resistant *Staphylococcus aureus* infections place a huge burden on the healthcare sector and the wider community. An increasing rate of infections caused by methicillin‐resistant *Staphylococcus aureus* (MRSA) has necessitated the development of alternative agents. We previously reported that usnic acid (UA) has activity against MRSA; here, we report the effect of UA in combination with norfloxacin on the drug resistance of MRSA clinical isolates. We observed that the combination of UA–norfloxacin significantly reduces the bacterial burden in mouse models infected with *S. aureus*, without causing any detectable associated toxicity. Proteomic analysis indicated that UA–norfloxacin induces oxidative stress within cells, which leads to membrane damage and inhibits metabolic activity and biosynthesis of peptidoglycan and fatty acids. Collectively, this study provides evidence that UA in combination with norfloxacin may be a potential candidate for development into a resistance‐modifying agent for the treatment of invasive MRSA infections.

## Abstract 2

This is the first report on the drug resistance‐modifying potential of usnic acid (UA) through inhibition of the multidrug resistance (MDR) efflux pump and down‐regulation of proteins involved in peptidoglycan and fatty acid biosynthesis. This compound may be helpful in the management of infection caused by MRSA through (a) lowering the prescribed amount of antibiotics, (b) decreasing MDR generation, and (c) intensifying the efficacy of antibiotics against MRSA/VRSA under both *in vitro* and *in vivo* conditions. These results may be helpful in the development of anti‐MRSA drug combinations from economical and non‐toxic natural products.

## Graphical abstract

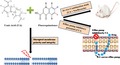

**Abbreviations**
CFU: colony forming units
CLSI: Clinical and Laboratory Standards Institute
CTC: 5‐cyano‐2,3‐di‐(*p*‐tolyl) tetrazolium chloride
EtBr: ethidium bromide
GAPDH: glyceraldehyde 3‐phosphate dehydrogenase
IPG: immobilized pH gradient
MALDI‐TOF/TOF: matrix‐assisted laser desorption/ionization time‐of‐flight/time‐of‐flight
MDR: multidrug resistance
MIC: minimum inhibitory concentration
MRSA: methicillin‐resistant *Staphylococcus aureus*
NO: nitric oxide
PI: propidium iodide
ROS: reactive oxygen species
UA: usnic acid
VRSA: vancomycin‐resistant *Staphylococcus aureus*

The infections caused by multidrug‐resistant pathogens pose a serious global public health challenge. The Centers for Disease Control and Prevention (CDC) has outlined the top 18 drug‐resistant threats, including methicillin‐resistant *Staphylococcus aureus* (MRSA), which require urgent attention to prevent the spread of infections 1. Recent reports from hospital and community surveillance studies together with the Infectious Diseases Society of America (ISDA) have termed the nosocomial infections the ‘ESKAPE pathogens’ (*Enterococcus faecium*,* Staphylococcus aureus*,* Klebsiella pneumonia*,* Acinetobacter baumannii*,* Pseudomonas aeruginosa* and *Enterobacter* species) 2, 3. *Staphylococcus aureus*, a Gram‐positive bacterium, is a ubiquitous member of the human microbiota and was recognized as a major pathogen in the late 19th century. It is the causative agent for a wide range of infections including skin infections, sepsis, toxic shock syndrome, endocarditis and osteomyelitis 4. This pathogen has gained wide attention due to the emergence of MRSA, vancomycin‐resistant *Staphylococcus aureus* (VRSA) and multidrug resistance (MDR) strains which have acquired resistance to agents of last resort including vancomycin and linezolid. This scenario presents an urgent need to develop alternative approaches to combat such infections, which include modulating drug plasma concentration within the therapeutic window 5 and use of immune modulation therapy and probiotics 6.

Plant‐derived natural products have gained global attention as novel antimicrobial compounds due to their being recognized as safe and their use in traditional medicine systems 7, 8. It has been well established that plant secondary metabolites possess antibacterial and resistance‐modifying properties with a possible novel mechanism of action 9, 10, 11 and simultaneously minimize the side effects associated with conventional antibiotics 12. Combination therapy has been successful in the treatment of drug‐resistant tuberculosis and extended‐spectrum β‐lactam‐producing Gram‐negative bacterial infections 13, 14, 15. Combination therapy includes two antibiotics to which the bacterial pathogens are susceptible in order to improve the clinical outcome with respect to monotherapy by broadening the antibacterial spectrum, synergistic effects and reduced risk of resistance development. Several studies have reported the synergistic interactions of many plant bioactive compounds with clinically used antibiotics to minimize the infection and also result in reduced rates of emergence of resistance 16, 17.

The bioactivity‐guided fraction of *Usnea subfloridana* led to the identification of usnic acid (UA), which has potent antibacterial activity against multidrug‐resistant clinical isolates of *S. aureus* with membrane damaging potential 18. The present study deals with the investigation of the synergistic interaction of UA with norfloxacin against clinical isolates of MRSA through *in vitro* and *in vivo* assays. A gel‐based proteomic approach was further employed to understand the possible mechanism of synergy.

## Materials and methods

### Bacterial strains and growth media

The details of clinical isolates used in the present study are presented in Table S1. All the bacterial strains were grown and maintained on Muller Hinton Agar (MHA)/Broth (MHB) at 37 °C. The inoculum was prepared in 0.85% NaCl and adjusted at 0.5 McFarland standard to obtain approximately 10^8 ^colony forming units (CFU)·mL^−1^.

### PCR amplification of *mecA* gene

All the eight clinical isolates of *S. aureus* were checked for the presence of the *mecA* gene, a marker for MRSA that impart resistance to β‐lactam antibiotics using PCR 19.

### Checkerboard assay for interaction study

The fractional inhibitory concentration (FIC) of UA with different antibiotics was quantified through a modified broth checkerboard method 20. The synergy between the antibiotics and UA was estimated using the fold reduction and fractional inhibitory concentration index (FICI) 21. The FIC was calculated as follows (where MIC is minimum inhibitory concentration):

$$\text{FIC}\left(\text{drug 1}\right)=\frac{\text{MIC of drug 1 in combination}}{\text{MIC of drug 1 on its own}}$$

$$\text{FIC}\left(\text{drug 2}\right)=\frac{\text{MIC of drug 2 in combination}}{\text{MIC of drug 2 on its own}}$$

FIC Index(FICI)=FIC(drug1)+FIC(drug2)

### Resistance studies

The propensity of bacteria to develop resistance and the mutation prevention concentration of the UA and norfloxacin combination was evaluated following methodology describe earlier 22, 23 using reference strain MTCC‐96 of *S*. *aureus*. First, the MIC of UA alone and in combination with norfloxacin was determined in MTCC‐96 as per the broth microdilution assay, in accordance with the Clinical and Laboratory Standards Institute (CLSI) guidelines. Following this, serial passaging was carried out by transferring the bacterial cells growing at sub‐MIC of the compound/antibiotic and subjecting them to another MIC assay. After 24 h of incubation, cells at sub‐MIC of the test compound/antibiotic were further transferred and assayed for MIC determination. The process was carried out for 20 passages and the fold increase in MIC was plotted against the number of passages to evaluate the propensity of the bacteria to develop resistance. The post‐antibiotic effect of UA alone and in combination with norfloxacin against the clinical isolate MRSA‐2071 was measured using a previously described method 24.

### Time–kill studies

To assess the bactericidal potential of UA and norfloxacin against MRSA‐2071, a time–kill kinetic study was performed at different concentrations alone and in combination as per the CLSI methods 25. Each analysis was carried out in triplicate and compared to untreated control. The criterion for a bactericidal effect was ≥ 3‐log_10_ decrease in CFU count at a specified time while a decline of less than 3‐log_10 _CFU·mL^−1^ was interpreted as bacteriostatic.

### *In vivo* efficacy

Swiss Albino mice (male, 5–6 weeks old, 22–25 g, *n* = 5 per group) were infected with 10^6^ CFU·mL^−1^ of *S. aureus* (MTCC‐96) through an intravenous route to evaluate therapeutic efficacy. The treatment groups comprised combinations (norfloxacin 8 mg·kg^−1^ + UA 1 mg·kg^−1^; norfloxacin 4 mg·kg^−1^ + UA 0.5 mg·kg^−1^) along with norfloxacin alone (1, 5, 10 and 16 mg·kg^−1^ body weight) and UA alone (0.5, 1 and 4 mg·kg^−1^ body weight). Oral administration of treatments once daily was initiated 16 h after infection and continued till day 7. Blood was collected from retro‐orbital plexus 24 h after the last dose for toxicity assessment and spleen to check the bacterial load 18. The bacterial count was finally expressed as CFU·g^−1^ tissue and presented as the mean ± SEM. The reduction in the bacterial load within the tissues was assessed by comparing the load in the treatment groups with respect to infection control.

### *In vivo* toxicity determination

*In vivo* toxicity in Swiss Albino mice was studied in accordance with the Organization for Economic Co‐operation and Development test guideline no. 423 with additional modifications 26. The animals were observed for body weight changes in addition to morbidity and mortality. In addition, serum from the blood was analyzed for various biochemical parameters pertaining to hematology, liver, and kidney functioning along with the lipid profile.

### Ethidium bromide accumulation and efflux

In order to evaluate the efflux pump inhibitory/modulatory potential of UA, spectrofluorometric determination of ethidium bromide (EtBr) efflux was performed as per the earlier reported method 27. Efflux inhibition was assessed by measuring the decline in fluorescence intensity of EtBr over a time of 30 min. Reserpine, an efflux pump inhibitor, was used as positive control. The experiments were performed thrice and the data were expressed as mean ± SEM. An EtBr accumulation assay was carried out using a flow cytometer (LSR II, BD Bioscience, San Diego, CA, USA) following the method described earlier 28. Further, the expression analysis of five different membrane‐associated efflux pump genes was evaluated upon exposure to UA and norfloxacin, alone and in combination.

### Membrane permeabilization assay

The effect of the UA–norfloxacin combination on membrane permeability in MRSA‐2071 was assessed following propidium iodide (PI) uptake method described earlier and the fluorescence was measured at excitation and emission of 544 nm and 620 nm, respectively 29 (FLUOStar Omega; BMG Labtech, Ortenberg, Germany). A negative control contained untreated MRSA‐2071 bacterial cells and PI with Milli‐Q water while cells treated with 2.8% formaldehyde and 0.04% glutaraldehyde served as positive control. Untreated cells which possess intact membrane and do not allow PI to intercalate with DNA and fluoresce served as a negative control. Formaldehyde and glutaraldehyde used as fixatives which destroy the integrity of the bacterial membrane and thus allow PI to bind to DNA served as a positive control.

### Cytoplasmic membrane depolarization assay

Membrane depolarization was studied in MRSA‐2071 using membrane‐potential‐sensitive cyanine dye diSC_3_‐5, according to the earlier reported method 30. MRSA‐2071 cells with mid‐logarithmic phase (*D*_600_ = 0.6) were collected and washed once with buffer (5 mm HEPES, pH 7.2, 5 mm glucose) and resuspended in same buffer to achieve the *D*_600_ = 0.05. The cell suspension was incubated with 0.4 μm diSC3‐5 until dye uptake was maximal (approximately 90%), after which 100 mm KCl was added to equilibrate the cytoplasmic and external K^+^ concentration. *Staphylococcus aureus* cells (*D*_600_ = 0.3) were treated with phytomolecules and carbonyl cyanide *m*‐chlorophenylhydrazone as a positive control at 37 °C. Culture without treatment served as a negative control. The fluorescence reading was monitored with a microplate reader (FLUOStar Omega) at an excitation wavelength of 622 nm and an emission wavelength of 670 nm. The blank with only cells and the dye was used to deduct the background. The experiments were performed thrice and data expressed as mean values ± SEM (**P < 0.01 *vs* control; *P* > 0.05 represents non‐significant: Dunnett's test).

### Proteomic studies to investigate the mechanism of action of UA–norfloxacin combination

The mode of anti‐staphylococcal activity of the UA–norfloxacin combination was determined through a gel‐based proteomic approach. Protein extraction was carried out from bacterial cells (MRSA‐2071) exposed to sub‐lethal concentrations of UA, norfloxacin and UA–norfloxacin combination using a TRIzol extraction protocol 31. Untreated MRSA‐2071 cells grown to the same attenuance served as control. Protein samples (225 μg) were used for passive rehydration of 17 cm immobilized pH gradient (IPG) strips (linear pH 4–7; Bio‐Rad, Hercules, CA, USA) overnight. A gradient of voltage starting from 200 V for 4 h (step and hold), 500 V for 1 h (step and hold), 1000 V for 1 h (step and hold), 8000 V for 13 500 Volt‐hour (gradient) and 8000 V for 8 h (step and hold), 500 V for 4 h (step and hold) and 200 V for 2 h (step and hold) resulting in a total of approximately 93 kV for isoelectric focussing (IEF) was used followed by resolution on 12.5% SDS/PAGE in triplicate. Coomassie brilliant blue stained images were imported to ht analyzer 2d (Genomic Solution, Ann Arbor, MI, USA) software for comparative analysis. Spots present in all the gels were considered for detailed multivariate analysis and differentially expressed spots were excised and stored at 4 °C for subsequent mass spectrometric analysis.

### In‐gel digestion and protein identification by MALDI‐TOF/TOF

In‐gel digestion of the excised spots was performed following the earlier described protocol 31, 32. The protein identification was performed with matrix‐assisted laser desorption/ionization time‐of‐flight/time‐of‐flight (MALDI‐TOF/TOF) mass spectrometry (UltrafleXtreme™ mass spectrometer; Bruker Daltonics Inc., Bremen, Germany). A combined MS and LIFT tandem mass spectrometry was performed using biotools 3.0 software (Bruker Daltonics Inc.). The TOF spectra were recorded in positive ion reflector mode with a mass range from 700 to 3500 Da. Only proteins matched by a minimum of two unique peptide sequences were included in the results list. Sequence coverage was also considered to evaluate protein identification.

### Functional pathway and network analysis

The differentially expressed proteins identified in two‐dimensional electrophoresis were subjected to DAVID (Database for Annotation, Visualization, and Integrated Discovery) database version 6.7 (https://david.ncifcrf.gov/) 33. The list of UniProt IDs of the proteins was further imported into KOBAS 3.0 (KEGG Orthology‐Based Annotation System) database and the pathways were mapped against *S. aureus* using default settings 34. In addition, a protein–protein interaction analysis was also carried out using the STRING v10.0 database (https://string-db.org/) with default parameters and *S. aureus* subsp. *aureus* as the organism of interest 35.

### Measurement of reactive oxygen species

The analysis of intracellular levels of reactive oxygen species (ROS) was carried out using the ROS‐sensitive probe CM‐H_2_DCFDA (Invitrogen, Carlsbad, CA, USA) as per the method described by Wang and Joseph 36. The ROS level was measured in terms of reduced fluorescent compound dichlorofluorescein (DCF) with excitation and emission wavelengths of 485‐10 nm and 520 nm, respectively, using a microplate reader (FLUOStar Omega). Ciprofloxacin served as a positive control, while untreated MRSA‐2071 cells served as a negative control in the study.

### Measurement of nitrite level

The nitrite level within the MRSA‐2071 cells exposed to UA and norfloxacin alone and in combination was quantified by spontaneous oxidation of nitric oxide (NO) 37 using a Griess reagent kit according to the manufacturer's protocol (Life Technologies, Camarillo, CA, USA). Sodium nitroprusside, a standard NO producer, was used as a positive control, while a reference sample was prepared by mixing Griess reagent and deionized water. The absorbance of the nitrite was measured at 548 nm using a microplate reader (FLUOStar Omega). The experiment was performed in triplicate and data expressed are mean values ± SEM (**P < 0.01 *vs* control: Dunnett's test).

### Metabolic activity using resazurin

A resazurin assay was performed in MRSA‐2071 cells exposed to UA and norfloxacin alone and in combination at different concentrations to check the cell viability and metabolic activity 38. Untreated MRSA‐2071 actively growing cells served as a positive control in the study. Resazurin dye, at a concentration 10 μg·mL^−1^, was used and the fluorescence intensity was measured at 590 nm for 30 min at 15 s intervals in a microplate reader (FLUOStar Omega). The experiment was performed in triplicate and data expressed are mean values ± SEM (**P < 0.01 *vs* control: Dunnett's test).

### Assessment of bacterial respiratory activity using 5‐cyano‐2,3‐di‐(*p*‐tolyl) tetrazolium chloride

Respiratory activity assay using 5‐cyano‐2,3‐di‐(*p*‐tolyl) tetrazolium chloride (CTC) was performed as described previously 39. MRSA‐2071 cells exposed to UA and norfloxacin alone and in combination at MIC concentrations for 4 h were used for the assay. CTC at the final concentration of 5 mm was added, followed by fixing the cells with 2.8% formaldehyde and 0.04% glutaraldehyde and counterstaining with 4′6‐diamidino‐2‐phenylindole. A negative control was prepared by disrupting the membrane prior to addition of CTC. Further, the samples were analyzed through flow cytometer using the FL‐1 channel (488 nm; blue argon laser) and cell quest 3.3 analysis software (LSR II, Becton‐Dickinson, San Diego, CA, USA).

### Western blot analysis

The overnight grown culture of MRSA‐2071 in MHB at 37 °C with agitation was diluted in MHB and again grown at 37 °C to a *D* of 0.6 with or without treatments. The cells were harvested, the pellet was washed and resuspended in TE buffer (10 mm Tris, pH 7.5, 1 mm EDTA) followed by sonication, and cell debris was removed by centrifugation at 11 269 ***g*** for 10 min at 4 °C. Following the estimation of proteins using the Bradford assay, aliquots of protein (80 μg) were loaded on 12.5% SDS/PAGE gels and blot transferred onto polyvinylidene difluoride membrane. After blocking with 5% skimmed milk, MurA was probed with anti‐MurA antibody at 1 : 2000 dilution, followed by incubation with secondary alkaline phosphatase conjugated goat anti‐rabbit antibody at 1 : 5000 dilution. The blot was further developed using nitro blue tetrazolium/5‐bromo‐4‐chloro‐3‐indolyl‐phosphate substrate with glyceraldehyde 3‐phosphate dehydrogenase (GAPDH) as an endogenous control.

### qRT‐PCR analysis

The qRT‐PCR analysis of identified genes was analyzed in MRSA‐2071 cells in the presence of UA and norfloxacin alone and in combination. The list of all the primers is presented in Table S4. Cells were grown to mid‐log phase in the presence of different treatments at sub‐lethal concentrations and real‐time quantification of RNA transcript was analyzed by SYBR GreenER qPCR mix (Invitrogen) using the 7900HT fast real‐time PCR system (Applied Biosystems, Foster City, CA, USA). Observations were recorded in terms of log RQ after normalization with GAPDH, an endogenous control.

### Ethical clearance

The protocols (CIMAP/IAEC/2016‐19/06 and CIMAP/IAEC/2016‐19/01) were duly approved by Institutional Animal Ethics Committee on 29 February 2016.

### Statistical analysis

The data were subjected to a one‐way ANOVA to analyze the mean values obtained for the treatment and control. Dunnett's test was used to compare the treatment and control and statistical significance was set at *P ≤ 0.05, **P ≤ 0.01 and ***P ≤ 0.001, and *P* > 0.05 was considered to be non‐significant.

## Results and Discussion

The extensive use of antibiotics has raised serious health concerns throughout the world due to the development and emergence of MRSA, Vancomycin Intermediate *Staphylococcus aureus*/ Vancomycin Resistant *Staphylococcus aureus* (VISA/VRSA) and MDR *S. aureus*, urging the need to identify new agents. Plant secondary metabolites have displayed a potential as antibacterial agents individually and are known to potentiate the activity of other antibiotics through different mechanisms and thus they could be employed for the treatment of resistant pathogens 40, 41, 42, 43, 44. UA, a dibenzofuran derivative from lichens, has already been established as having antibacterial potential against MRSA with the probable mode of action being its membrane disruptive property 18, 45. However, no detailed reports are available for the resistance‐modifying potential of UA in combination with conventional antibiotics against MRSA. The present comprehensive study describes the synergistic interaction of UA with norfloxacin against clinical isolates of *S. aureus* (MRSA), which were found to be multidrug resistant and harbor *mec*A gene (Table S2 and Fig. S1). In order to assess the utility of UA (particularly in combination) as a therapeutic agent, the propensity of bacteria (MRSA‐2071) to acquire resistance against UA alone as well as in combination with norfloxacin was evaluated (Fig. S2a). The results indicate that clinical strains of *S. aureus* (MRSA‐2071) were not able to develop resistance against UA alone as well as in combination with norfloxacin even after 20 subsequent passages. A 2‐ to 4‐fold increase in the MIC of UA alone was observed after 12–20 subsequent passages. However, in the case of norfloxacin alone, more than 900‐fold increase in MIC was observed during the same period. The prolongation of the post‐antibiotic effect of norfloxacin in the presence of UA represents a continued suppression of growth after a short exposure to the antimicrobial agents. Also, UA not only increased the inherent susceptibility of *S. aureus* to norfloxacin, but also simultaneously reduced the emergence of norfloxacin resistance (Table S3, Fig. S2b). All these results collectively suggest the suitability of UA in combination with norfloxacin. A similar observation has been made using an isoliquiritigenin derivative (IMRG4), a natural plant flavonoid 46, suggesting the therapeutic potential of the UA–norfloxacin combination for treating MDR infections with delayed resistance.

### *In vitro* combination study

Synergy and the resistance‐modifying potential of UA with clinically used antibiotics were investigated and are presented in terms of fold reduction and FICI (Table 1). In combination with oxacillin and tetracycline, UA displayed synergy with 4–16‐fold reduction in MIC against all the tested clinical isolates. However, the UA–vancomycin combination showed synergistic interaction against four of the eight isolates (FICI 0.374–0.49) and the remaining four exhibited additive interaction with FICI 0.5–0.75. As evident from the results, UA could cause significant reduction of up to 16‐fold in the MIC of norfloxacin with FICI ≤ 0.50 and thus it was selected for further studies. The clinical breakout point for the susceptibility of norfloxacin is ≤ 10 mg·L^−1^ 47, and its MIC in combination with UA was observed to reach close to the susceptible limit as defined in the European Committee on Antimicrobial Susceptibility Testing guidelines (31.25 mg·L^−1^).

**Table 1:** *In vitro* anti‐staphylococcal activity of UA with different antibiotics against methicillin‐resistant clinical isolates of *Staphylococcus aureus*. FICI, fractional inhibitory concentration index; FR, fold reduction in MIC of antibiotics; MIC, minimum inhibitory concentration; NOR, norfloxacin; OXA, oxacillin; TET, tetracycline; UA, usnic acid; VAN, vancomycin

Strains	MIC of UA alone (μg·mL^−1^)	MIC of antibiotics alone (μg·mL^−1^)				MIC of OXA in presence of UA (μg·mL^−1^)		MIC of NOR in presence of UA (μg·mL^−1^)		MIC of VAN in presence of UA (μg·mL^−1^)		MIC of TET in presence of UA (μg·mL^−1^)	
		OXA	NOR	VAN	TET	MIC	FICI (FR)	MIC	FICI (FR)	MIC	FICI (FR)	MIC	FICI (FR)
SA‐2071	25	1000	500	3.125	25	15.6	0.265 (64)	31.25	0.31 (16)	0.78	0.49 (4)	3.125	0.25 (8)
SA‐1745	25	1000	500	6.125	50	31.25	0.281 (32)	62.5	0.375 (8)	1.56	0.379 (4)	6.25	0.375 (8)
SA‐5944	25	1000	500	1.56	12.5	31.25	0.28 (32)	125	0.375 (4)	0.39	0.5 (4)	3.125	0.37 (4)
SA‐4627	25	500	250	1.56	50	62.5	0.25 (8)	62.5	0.312 (8)	0.39	0.5 (4)	12.5	0.31 (4)
SA‐4423	50	500	250	1.56	25	62.5	0.375 (8)	62.5	0.37 (8)	0.39	0.375 (4)	6.25	0.3 (4)
SA‐4620	25	1000	250	1.56	25	62.5	0.31 (16)	31.25	0.375 (8)	0.78	0.75 (2)	3.125	0.375 (8)
SA‐3151	25	500	500	1.56	25	31.25	0.31 (16)	62.5	0.325 (8)	0.195	0.625 (8)	6.25	0.31 (4)
SA‐10760	50	250	500	3.125	50	62.5	0.375 (4)	125	0.49 (4)	0.78	0.374 (4)	6.25	0.312 (8)

### *In‐vivo* anti‐staphylococcal activity and toxicity

**Figure 1:**
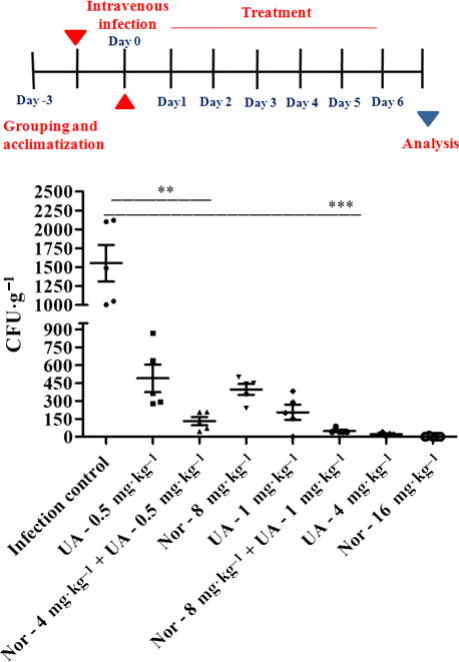
*In vivo* anti‐staphylococcal efficacy of UA, norfloxacin (Nor) and UA–norfloxacin combination at various doses in terms of reduction of bacterial burden (*Staphylococcus aureus* MTCC‐96) in spleen tissue. The infection (1 × 10^6^ CFU·mL^−1^ in 200 μL) was induced through the intravenous route. The bacterial load (CFU·g^−1^) of spleen tissue was expressed as mean ± SEM. (*P ≤ 0.05, **P ≤ 0.01, ***P ≤ 0.001, Dunnett's test.)

The bacterial load in spleen tissue of Swiss Albino mice was determined to evaluate the *in vivo* efficacy of the UA–norfloxacin combination (Fig. 1). A dose‐dependent significant reduction was observed where 25% of the effective dose (ED_25_; 0.5 mg·kg^−1^) of UA in combination with ED_25_ (4 mg·kg^−1^) of norfloxacin could reduce the bacterial load up to 50% (*P *<* *0.01), whereas up to 80% (*P *<* *0.001) reduction was recorded when the UA–norfloxacin combination was administered at 50% of the effective dose (ED_50_; Nor 8 mg·kg^−1^ and UA 1 mg·kg^−1^). Toxicity studies were also performed to evaluate the effect of the UA–norfloxacin combination on healthy animals and animals infected with MTCC‐96. No lethal effects were recorded post‐treatment with the combination in the biochemical parameters compared to vehicle control, with and without infection (Table 2). The results further provide evidence for the low toxicity and acceptable safety profiles in mice models for therapeutic use. These are in accordance with previous reports describing the efficiency of plant molecules in combination with conventional antibiotics in reducing the bacterial load in animal models 48, 49.

**Table 2:** Hematological and biochemical changes recorded during the toxicity study in Swiss Albino mice. Values are mean ± SD. ALKP, alkaline phosphatase; RBC, red blood cells; SGOT, serum glutamate–oxaloacetate transaminase; SGPT, serum glutamate–pyruvate transaminase; WBC, white blood cells.

Parameters	Uninfected		Infected with MTCC‐96	
	Control	UA + Nor	Control	UA + Nor
Change in body weight (g)	3.64 ± 0.38	4.36 ± 0.44	−3.33 ± 0.31	−2.26 ± 0.27
Relative organ weight				
Liver	1.453 ± 0.19	2.115 ± 0.24	1.414 ± 0.27	1.178 ± 0.28
Spleen	0.556 ± 0.42	1.152 ± 0.30	1.257 ± 0.22	1.097 ± 0.5
SGPT (U·L^−1^)	7.15 ± 0.54	6.95 ± 0.63	6.835 ± 0.49	6.874 ± 0.31
SGOT (U·L^−1^)	10.96 ± 0.66	11.08 ± 0.38	12.36 ± 0.74	13.05 ± 0.63
Serum creatinine (mg·dL^−1^)	0.14 ± 0.08	0.17 ± 0.27	0.15 ± 0.18	0.16 ± 0.11
Serum ALKP (U·L^−1^)	118.04 ± 3.18	120.27 ± 2.21	114.39 ± 1.76	117.64 ± 2.91
Serum total cholesterol (mg·dL^−1^)	61.42 ± 2.09	57.92 ± 2.16	63.37 ± 1.88	65.69 ± 3.13
Serum bilirubin (mg·dL^−1^)	0.07 ± 0.017	0.06 ± 0.005	0.07 ± 0.004	0.08 ± 0.013
Serum triglycerides (mg·dL^−1^)	28.74 ± 3.74	31.36 ± 2.58	29.49 ± 2.14	31.33 ± 1.76
RBC (millions·mm^−3^)	4.532 ± 0.56	5.971 ± 0.29	6.059 ± 0.4	6.114 ± 0.28
WBC (millions·mm^−3^)	8.15 ± 0.56	12.22 ± 0.49	11.4 ± 0.27	12.23 ± 0.53
Hemoglobin (g·dL^−1^)	12.96 ± 0.35	12.14 ± 0.28	11.175 ± 1.04	11.54 ± 0.82

### Killing kinetics

A dose‐ and time‐dependent decline in viable MRSA‐2071 cells was observed upon exposure to the UA–norfloxacin combination. The bactericidal effect of norfloxacin was achieved at a very low concentration in combination with UA (Fig. S3). Similar observations have also been reported earlier wherein plant molecules in combination with conventional antibiotics have been found to reduce the dose of partner antibiotics by many fold irrespective of their own activity 50.

### EtBr accumulation and efflux

**Figure 2:**
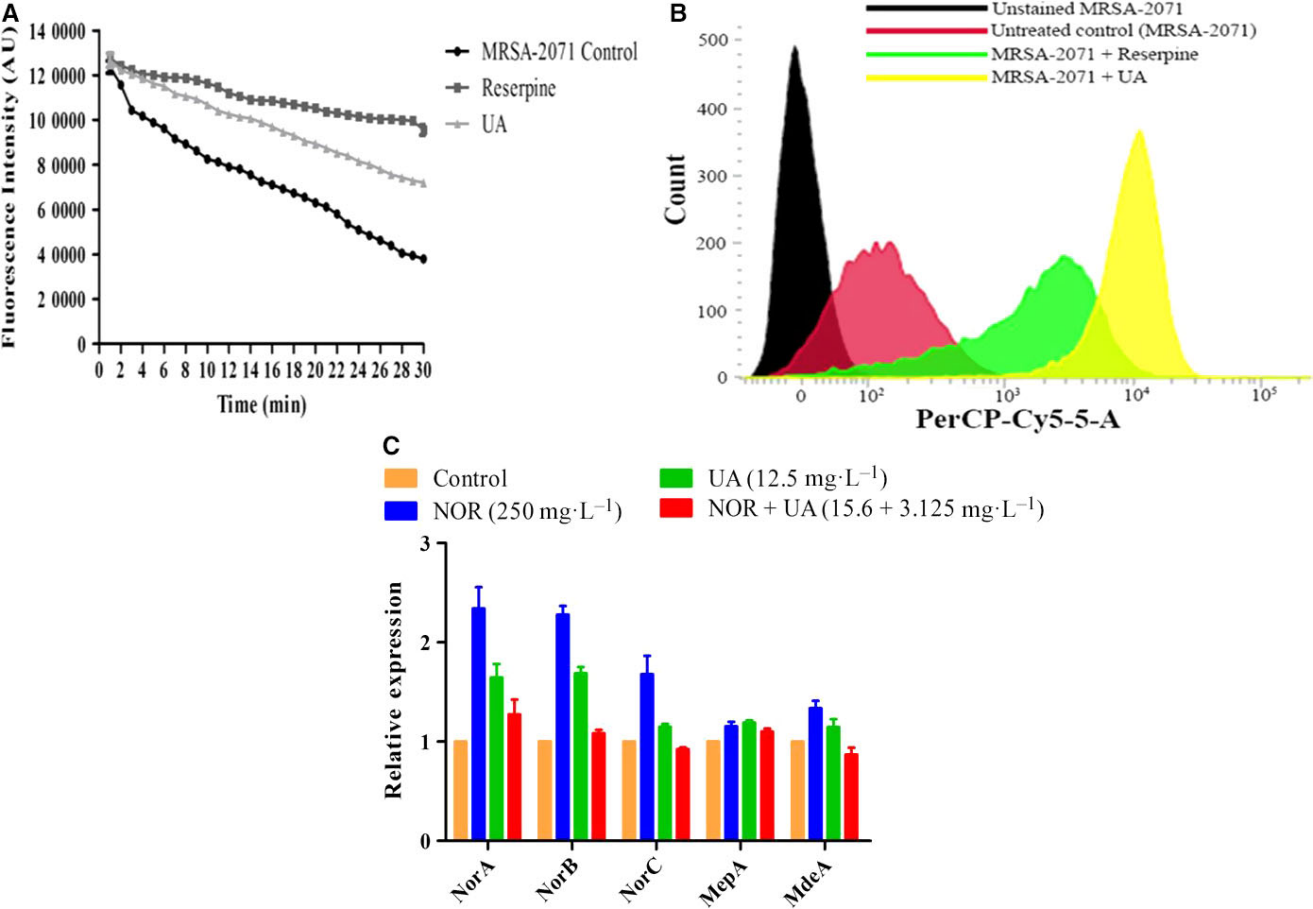
(A,B) Efflux pump modulatory potential of UA in clinical isolate MRSA‐2071 using EtBr as marker, determined through spectrofluorometric analysis (A) and flow cytometric analysis (B). Reserpine, an efflux pump inhibitor, was used as positive control in both the experiments. (C) Expression analysis of efflux pump genes in the presence of UA alone and in combination with norfloxacin.

Increased efflux is one of the major mechanisms which prevents antimicrobial agents from reaching their targets and contributes to the MDR phenotypes of pathogens 27. The efflux pump inhibitory potential of UA was evaluated using a spectrofluorometric assay, displaying a decline in EtBr efflux over a time duration of 30 min in MRSA‐2071 cells exposed to UA as compared to untreated cells. UA demonstrated an efflux pump inhibitory potential in MRSA‐2071 cells, comparable to that of reserpine (Fig. 2A). Flow cytometric analysis displayed a 1.8‐fold shift in the intensity of red fluorescence inside the cells in the presence of UA as compared to untreated control. In the presence of reserpine, however, a 1.2‐fold reduction in the intensity of red fluorescence was observed (Fig. 2B). The overexpression of efflux pump genes *norA*,* norB*,* norC*,* mepA* and *mdeA*, which are implicated in conferring multidrug resistance 51, upon norfloxacin exposure obtained in this study are in accordance with previous reports 52, 53. However, the down‐regulation of these in the presence of UA alone and further reduced expression in case of the UA–norfloxacin combination clearly demonstrate the efflux pump inhibitory potential of UA in MRSA (Fig. 2C). Collectively, these results indicate that UA modulates the activity of the MDR efflux pump of MRSA‐2071.

### Alteration of membrane permeability and potential

**Figure 3:**
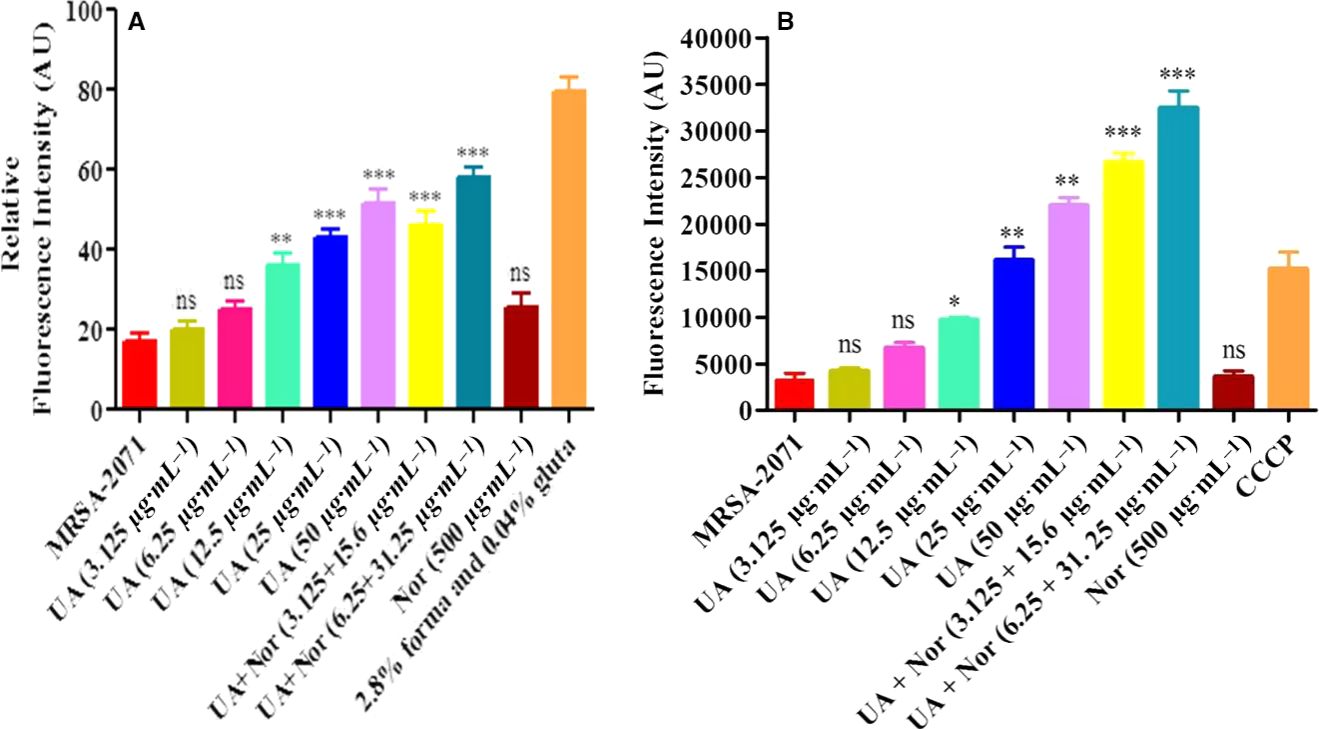
Prospecting membrane disruptive property of UA–norfloxacin combination against clinical isolate MRSA‐2071. (A) Membrane permeabilization using PI through spectrofluorimetry. Data represent mean ± SEM of three independent experiments (**P ≤ 0.01, ***P ≤ 0.001, Dunnett's test). (B) Dissipation of membrane potential by increase in the fluorescence of DiSC_3_‐(5) with the treatment of UA and norfloxacin alone as well as in combination at different concentrations (*P ≤ 0.05, **P ≤ 0.01, ***P ≤ 0.001, Dunnett’s test). Forma; Formaldehyde; Gluta; Glutaraldehyde.

The effect of the UA–norfloxacin combination on membrane permeability and potential was determined using spectroscopic techniques. Notably, MRSA‐2071 cells exposed to UA alone and the UA–norfloxacin combination displayed an increase in PI uptake as compared to untreated control (Fig. 3A) indicating altered membrane permeability. DiSC_3_‐5 is well known to intercalate into the cytoplasmic membrane of energized cells leading to quenching of the initial fluorescence, and increased fluorescence highlights the disruption in the membrane potential 54, 55. The UA–norfloxacin combination was found to dissipate the membrane potential to a much higher extent in comparison to UA and norfloxacin alone (Fig. 3B). All these results collectively demonstrate the membrane‐damaging ability of the UA–norfloxacin combination in MRSA‐2071.

### Modulation of pathways and networks in MRSA‐2071 due to UA–norfloxacin exposure revealed by gel‐based proteomic analysis

Multivariate analysis between the proteome of MRSA‐2071 control and the UA–norfloxacin combination (0.5 MIC – 3.125 + 15.6 mg·L^−1^) indicated the presence of around 1200 spots among which 34 spots exhibited differential expression (*P *≤* *0.05). The gel image with identified proteins is presented in Fig. S4. The protein identification details obtained from the MALDI‐TOF/TOF analysis are presented in Table 3. DAVID, PANTHER and STRING databases were used to classify and identify the potential function of altered proteins within the cells (Figs S5, S6). A cluster of proteins involved in oxidative stress responses was found to be up‐regulated in response to the UA–norfloxacin combination in MRSA‐2071 cells, which is in accordance with the earlier report where similar results were obtained in MRSA cells exposed to sub‐inhibitory concentrations of UA 44. Oxidants such as ROS and NO affect the viability of cells and hinder the process of pathogenesis 56 by irreversible oxidative damage to DNA, lipids and proteins 57, 58 and bring the tissues to homeostasis 59, 60. The treatment further decreased the expression of some of the proteins involved in peptidoglycan and fatty acid biosynthesis. In particular, a 6.64‐fold decline was observed in the expression level of UDP‐*N*‐acetylglucosamine 1‐carboxyvinyltransferase (MurA), which catalyzes the first step in peptidoglycan biosynthesis. In addition, four proteins which were uniquely identified in the cells exposed to the UA–norfloxacin combination at sub‐inhibitory concentrations were mainly involved in transcriptional regulation, namely DEAD‐box ATP‐dependent RNA helicase CshA, transcriptional regulatory protein WalR, elongation factor Tu and UPF0355 protein MRSA252. Moreover, sequence level in‐depth analysis of the uncharacterized protein UPF0355 identified uniquely in the combination‐treated cells displayed a putative role in modulating the operon regulating the downstream expression of genes involved in oxidative stress responses. Another remarkable observation was a 0.95‐fold down‐regulation of d‐lactate dehydrogenase, encoded by *ldhD*, which has been implicated in imparting vancomycin resistance in *S. aureus* by incorporating d‐lactate at the terminal residue of peptidoglycan precursors 61, 62. Another key observation in cells exposed to the UA–norfloxacin combination was the down‐regulation of tetracycline resistance protein tetM from transposon Tn5251 (TetM), implicated in imparting resistance to tetracycline. The above observations clearly point towards a resistance‐modifying ability of the UA–norfloxacin combination. Fatty acid biosynthesis is the first stage of membrane lipid biogenesis, playing a vital role in bacterial physiology 63, 64. FabI, unique to prokaryotes and a key regulator in controlling the elongation of the acyl chain for saturated fatty acids 65, 66, has been identified as a promising drug target 61. In the present study, the UA–norfloxacin combination was observed to down‐regulate the expression of enoyl‐(acyl‐carrier‐protein) reductase (NADPH) (FabI) as well as the initiation condensation enzyme 3‐oxoacyl‐(acyl‐carrier‐protein) synthase 3 encoded by *fabH* which may lead to inhibition of fatty acid biosynthesis.

**Table 3:** List of differentially expressed proteins in methicillin resistant *Staphylococcus aureus* SA‐2071 upon UA–norfloxacin exposure at sub‐lethal concentrations obtained from two‐dimensional electrophoresis analysis. D, down‐regulated; U, up‐regulated

Spot no.	Name of protein	Uniprot ID	Gene	*M* (Da)	No. of peptides	Fold change	Score	*P*‐value
U1	Superoxide dismutase (Mn/Fe)	P99098	*sodA*	22 711	12	0.27	243	0.004
U2	Alkyl hydroperoxide reductase subunit C	P99074	*ahpC*	20 977	8	0.33	217	0.005
U3	Enolase	P99088	*eno*	47 117	23	0.29	332	0.008
U4	Catalase	Q7A5T2	*katA*	58 380	27	0.23	207	0.009
U5	Probable quinol oxidase subunit 2	Q7A698	*qoxA*	41 777	24	0.31	199	0.016
U6	Probable thiol peroxidase	P99146	*tpx*	18 019	6	0.41	151	0.024
U7	Bacterial non‐heme ferritin	Q7A4R2	*ftnA*	19 589	5	0.49	148	0.027
U8	Protein GrpE	P99086	*grpE*	24 008	10	0.61	97	0.001
U9	Iron–sulfur cluster repair protein ScdA	Q7A7U6	*scdA*	25 485	17	0.35	226	0.019
U10	Transketolase	P99161	*tkt*	72 317	22	0.24	316	0.013
U11	Chaperone Protein DnaK	P99110	*dnaK*	66 361	20	0.35	208	0.024
U12	Peroxide responsive repressor (PerR)	Q7A4T8	*perR*	17 183	4	0.37	114	0.003
U13	UPF0413 protein SAUSA300_0983	Q2FI75	*SAUSA300_0903*	31 364	12	0.29	328	0.004
U14	Putative 2‐hydroxyacid dehydrogenase SAR2389	Q6GEC9	*SAR2389*	34 675	15	0.15	175	0.004
D1	Fructose‐bisphosphate aldolase Class I	P99117	*fda*	32 913	13	1.37	266	0.043
D2	d‐Lactate dehydrogenase	P99116	*ldhD*	36 756	18	0.95	254	0.036
D3	LipidII: glycine glycyltransferase	Q7A447	*femX*	48 513	21	1.48	310	0.001
D4	Putative antiporter subunit nnhG2	Q7A722	*mnhG2*	16 371	9	1.22	163	0.027
D5	DNA translocase FtsK	P64165	*ftsK*	90 682	26	1.06	336	0.013
D6	Trigger factor	P99080	*tig*	48 609	15	1.69	154	0.032
D7	UDP‐*N*‐acetylmuramoyl tripeptide–d‐alanyl‐d‐alanine ligase	A0A0H3JMW3	*murF*	50 065	8	3.53	186	0.007
D8	Enoyl‐(acyl‐carrier‐protein) reductase (NADPH)	A0A0H3JLH9	*fabI*	27 992	4	1.98	119	0.004
D9	3‐Oxoacyl‐(acyl‐carrier‐protein) synthase 3	P99159	*fabH*	33 879	11	1.96	176	0.023
D10	30S Ribosomal protein S12	P0A0G8	*rpsL*	15 287	9	1.88	143	0.018
D11	Uncharacterized lipoprotein SAUSA300_0411	Q2FJK3	*SAUSA300_0411*	30 917	10	1.52	281	0.047
D12	Tetracycline resistance protein tetM from transposon Tn5251	Q54807	*tetM(5251)*	72 556	16	2.15	294	0.024
D13	Deoxyribose phosphate aldolase 2	P99174	*deoC2*	23 218	14	1.36	173	0.022
D14	Formate acetyltransferase	Q7A7X6	*pflB*	84 862	23	1.73	304	0.021
D15	Adenine phosphoribosyltransferase	P68779	*apt*	19 117	12	1.84	265	0.03
D16	Fructose‐bisphosphate aldolase Class I	P99117	*fda*	32 913	16	1.34	208	0.005
D17	Fructose‐bisphosphate aldolase Class II	P99075	*fba*	30 836	18	1.42	314	0.014
D18	ATP synthase subunit c	Q7A4E6	*atpE*	6979	3	1.26	101	0.029
D19	HTH‐type transcriptional regulator SarR	Q7A425	*sarR*	13 669	5	1.9	151	0.006
D20	UDP‐*N*‐acetylglucosamine 1‐carboxyvinyltransferase	P84058	*murA*	45 054	15	6.64	163	.012
1^a^	60 kDa chaperonin	P99083	*groEL*	57 630	13	n/a	138	0.034
2^a^	General stress protein	W8U554	*yugI_1*	14 723	7	n/a	127	0.005
1^b^	50S ribosomal protein L17	Q7A469	*rplQ*	13 529	5	n/a	134	0.014
1^c^	DEAD‐box ATP‐dependent RNA helicase CshA	Q7A4G0	*cshA*	56 942	20	0	193	0.023
2^c^	Transcriptional regulatory protein WalR	Q7A8E1	*walR*	27 192	13	0	269	0.016
3^c^	Elongation factor Tu	P99152	*tuf*	43 104	15	0	338	0.018
4^c^	UPF0355 protein MRSA252	Q6GJR0	*SAR0405*	15 051	6	0	193	0.026

^a^ Uniquely expressed in only UA‐treated cells. ^b^ Uniquely identified in norfloxacin‐exposed cells. ^c^ Unique in UA–norfloxacin‐treated cells).

### Validation of proteomic signatures through cell‐based assays

**Figure 4:**
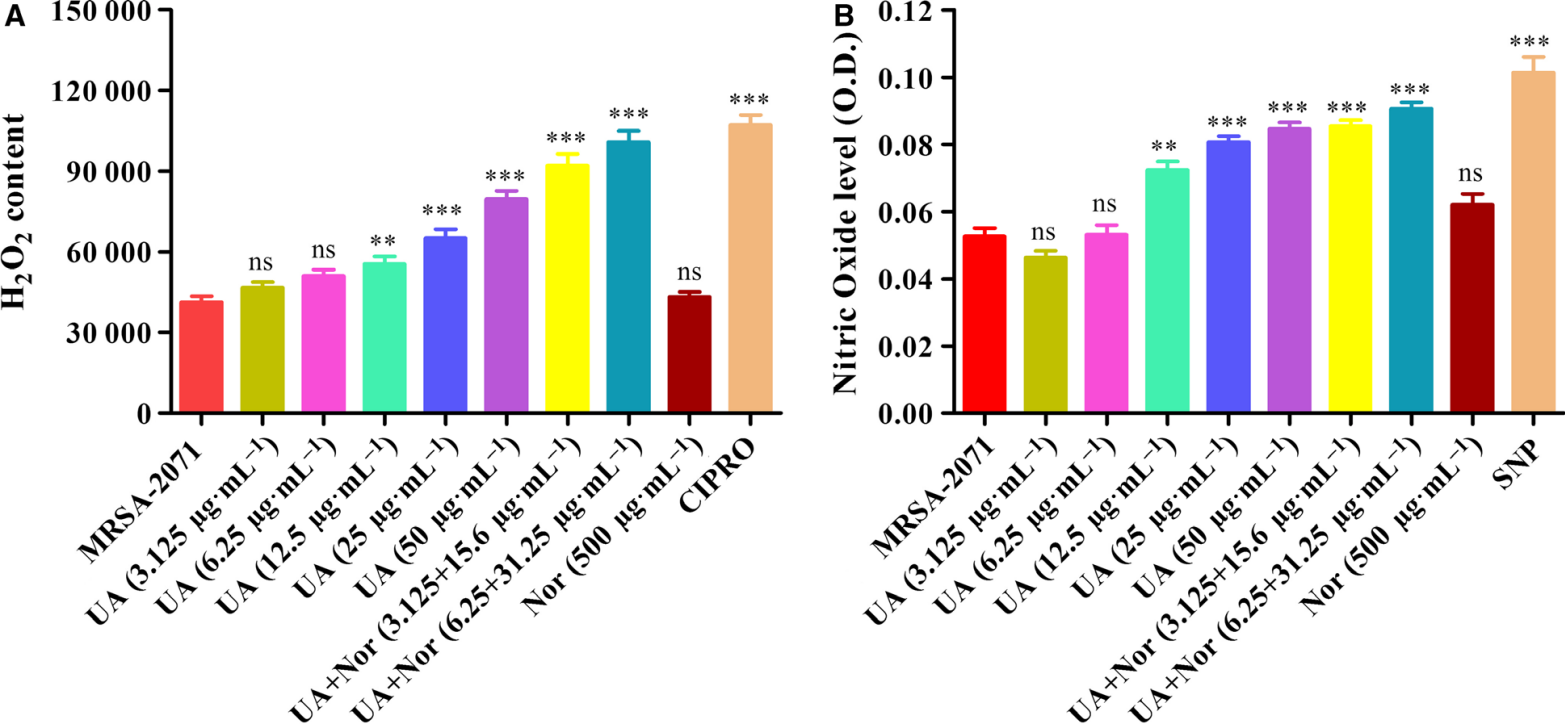
Effect of UA–norfloxacin combination on generation of ROS (H_2_O_2_ and NO). (A) Measurement of intracellular H_2_O_2_ levels in MRSA‐2071 exposed to different concentrations of UA and norfloxacin alone as well as in combination. Ciprofloxacin was used as a positive control in the study. (B) Effect of UA–norfloxacin combination at variable concentrations on generation of NO levels. Sodium nitroprusside was included in the study as positive control. The tests were performed thrice and data are expressed as mean ± SEM (ns, not significant; **P < 0.01, ***P < 0.001, Dunnett's test).

**Figure 5:**
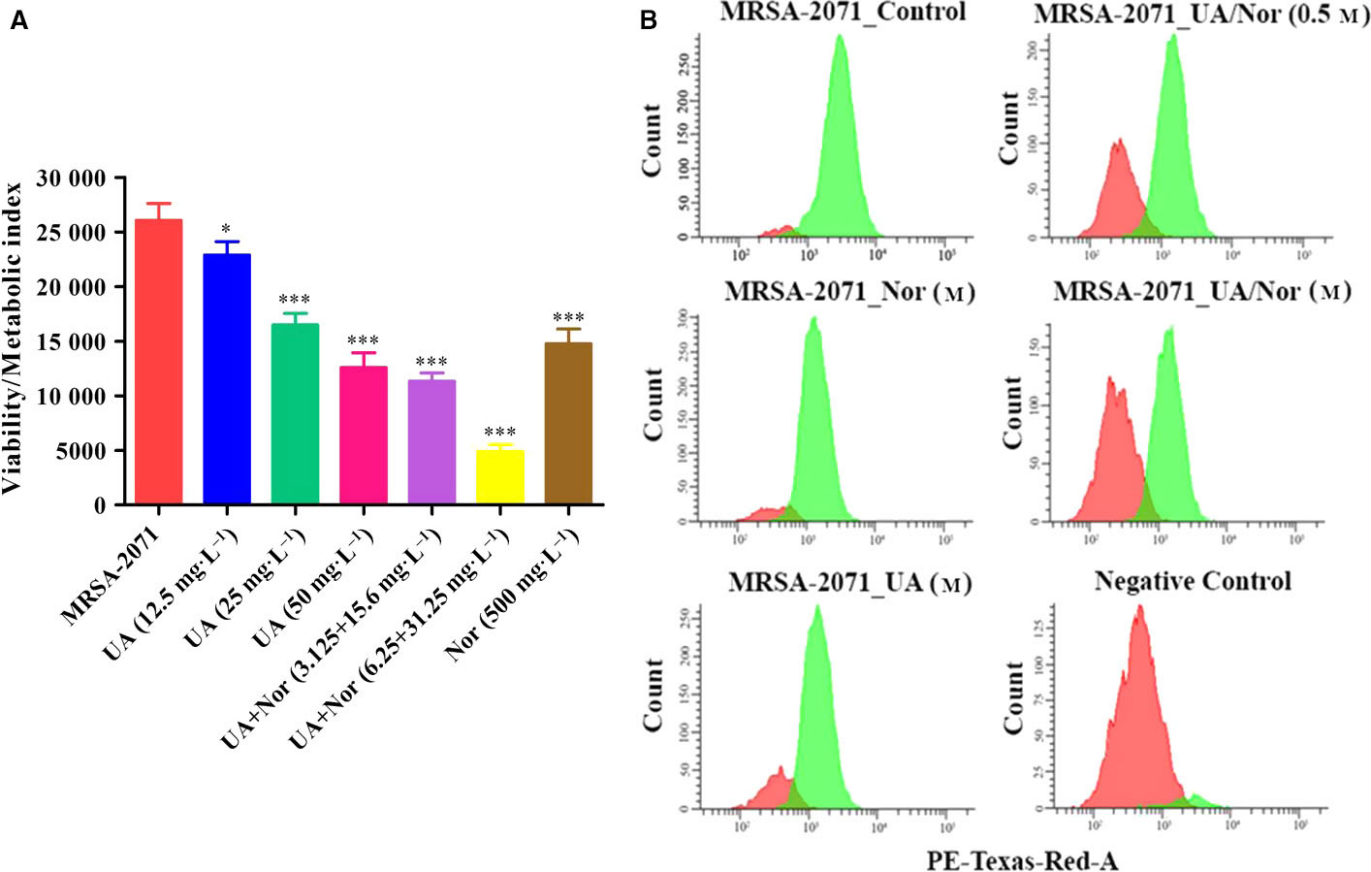
(A) Metabolic activity assay using resazurin. A gradual decline in metabolic activity of MRSA‐2071 cells was recorded with various concentrations of UA alone and the decrease was most prominent in the cells exposed to MIC concentrations of UA–norfloxacin combinations. The results are mean ± SEM derived from three independent experiments (*P ≤ 0.05, ***P ≤ 0.001 Dunnett's test). (B) Flow cytometric analysis for respiratory activity. Graphical representation of cell count *vs* CTC mean intensity (phycoerythrin‐Texas‐Red‐A) obtained in fluorescence‐activated cell sorting analysis of MRSA‐2071 control and treated samples. A dose‐dependent significant reduction in mean CTC intensity was observed compared to untreated control (*n* = 3). Negative control was prepared by fixing the cells with formaldehyde and glutaraldehyde prior to CTC staining.

**Figure 6:**
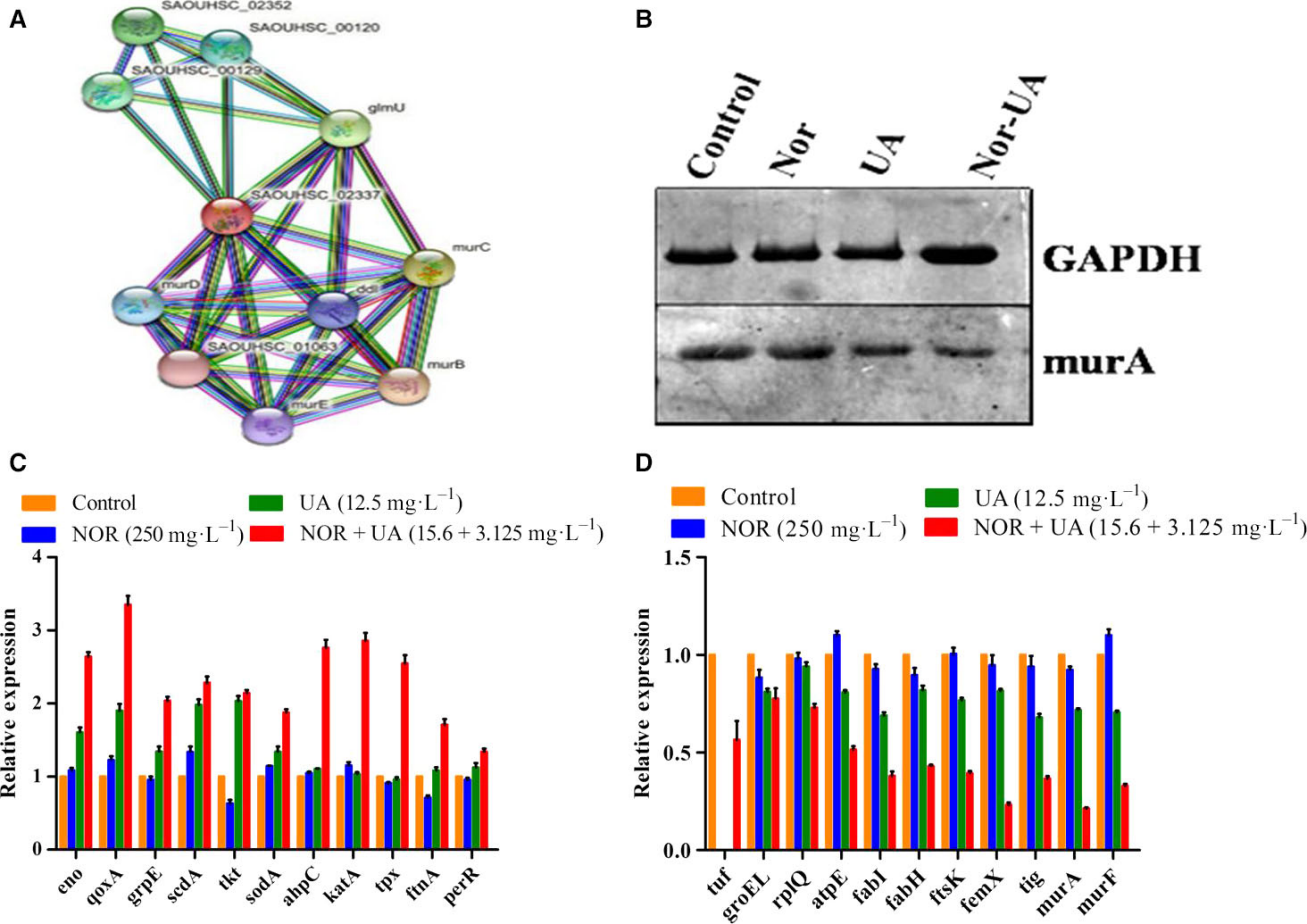
(A) Interactions of MurA with other proteins identified using STRING v.10.0 database. MurA, involved in catalyzing the first step in peptidoglycan biosynthesis, displayed strong interactions with proteins regulating peptidoglycan and cell‐wall biogenesis. (B) Western blot analysis of MurA. The alteration of MurA protein in response to UA–norfloxacin treatment was validated using western blotting, which confirmed the down‐regulation of MurA, indicated by lowered band width and intensity. GAPDH, an endogenous control, was used as loading control in the experiment. (C,D) Quantitative real‐time PCR analysis relative mRNA expression levels of genes up‐regulated in MRSA‐2071 (C) and down‐regulated (D) under UA–norfloxacin treatment alone and in combination using RT‐PCR. The relative expression analysis of each gene was carried out by normalizing the data with GAPDH as an endogenous control and the error bars represent standard deviation (*n* = 3).

Upon the exposure to the UA–norfloxacin combination, a considerable increase in H_2_O_2_ and NO levels was observed which was significantly higher compared to UA alone (Fig. 4). The present study for the first time reports the ROS‐mediated anti‐staphylococcal activity and resistance‐modifying potential of the UA–norfloxacin combination against a clinical isolate MRSA‐2071 (Indian origin). The up‐regulation of genes involved in mitigation of oxidative stress indicates that the UA–norfloxacin combination induces oxidative stress which could be detrimental to the cell and thus provides the basis for proposing it as an antibacterial and resistance‐modifying agent (see Fig. 6C). Functional assays clearly indicated decreased metabolic and respiratory activity upon exposure to the UA–norfloxacin combination (Fig. 5). These results also validate the proteomic data where enzymes involved in metabolic processes were found to be down‐regulated. Additionally, the down‐regulation of MurA, which is also linked to rest of the Mur proteins involved in successive steps of peptidoglycan biosynthesis and cell division (Fig. 6A), was further validated using immunoblotting and RT‐PCR, which clearly indicate the downregulation of MurA in UA–norfloxacin‐exposed cells (Fig. 6B). The RT‐PCR results were also in accordance with the proteomics data (Fig. 6C,D) where the *mur*A transcripts followed a similar pattern of down‐regulation in the cells exposed to the UA–norfloxacin combination. The altered membrane potential and permeability, decreased expression of fatty acid biosynthesizing enzymes and disruption in cellular respiration collectively validate the membrane‐damaging potential of UA in combination with norfloxacin.

The experiments were carried out in other clinical isolates as well to ensure the reproducibility of the results obtained with MRSA‐2071 (Figs S7–S10) .

In summary, this is the first comprehensive study describing the resistance‐modifying potential of UA in clinical isolate MRSA‐2071. The initial assays showed that UA acts synergistically with norfloxacin and inhibits the efflux pumps, one of the major processes involved in multidrug resistance. Further, the detailed mechanism of action was explored, which revealed that the UA–norfloxacin combination acts by perturbing multiple vital pathways essential for the bacteria to survive and persist. The efficacy and safety profile in the murine mouse model provide evidence the combination as a therapeutic agent against MRSA infections. The detailed analysis evidently demonstrates the effect of UA on inhibition of efflux pumps, followed by induction of oxidative stress, down‐regulation of peptidoglycan and fatty acid biosynthesis, altered membrane potential, perturbed respiration and metabolic activity. The UA–norfloxacin combination was found to further augment these observations as compared to UA alone.

## Conflict of interest

The authors declare no conflict of interest.

## Author contributions

SS and MPD conceived and designed the study. SS, VG, PK and RJ performed the experiments. SS, RK, AP and MPD analyzed the data. SS, AP and MPD wrote the manuscript.

## Supporting information


**Table S1.** Source of clinical isolates used in the present study.
**Table S2**. Antibiotic sensitivity/resistance profiling of clinical isolates of *S. aureus*.
**Table S3.** Mutation frequency of *S. aureus* (MTCC‐96) with UA and norfloxacin.
**Table S4.** List of primers used in the present study.
**Fig. S1.** PCR amplification of *mecA* gene from the clinical isolates of *S. aureus*. (Lane 1: SA‐2071, Lane 2: SA‐1745, Lane 3: SA‐5944, Lane 4: SA‐4627, Lane 5: SA‐3151, Lane 6: SA‐4423, Lane 7: SA‐10760, Lane 8: SA‐4620, Lane 9: MTCC SA‐96, Lane 10: Ladder.)
**Fig. S2.** (a) PAE of norfloxacin alone as well as in combination with UA against clinical isolate MRSA‐2071. (b) Propensity of development of resistance of *S. aureus* against UA and norfloxacin combination compared to norfloxacin alone.
**Fig. S3.** Time kill kinetics of MRSA‐2071 at different concentrations of UA and norfloxacin alone as well as in combination. These data represent mean ± SEM of three independent experiments.
**Fig. S4.** (a) Protein expression profiles of soluble proteins from MRSA‐2071 cells exposed to sub‐lethal concentrations of UA and norfloxacin alone as well as in combination. Proteins were resolved on 17 cm pH 4–7 IPG strips and 12.5% 20 cm SDS/PAGE gels. (½ MIC UA‐ 12.5 mg·L^−1^; ½ MIC Nor‐ 250 mg·L^−1^; ½ MIC UA/Nor‐ 3.125 + 15.6 mg·L^−1^). (b) Temporal proteome changes of MRSA‐2071 under UA–norfloxacin treatment identified using classical two‐dimensional electrophoresis. Proteins were separated in first dimension isoelectric focussing on 17 cm IPG strips of pH 4–7 range followed by second dimension separation on 12.5% SDS/PAGE. Multivariate analysis was performed to identify the differentially expressing protein spots in response to UA–norfloxacin treatment compared to untreated control and UA and norfloxacin alone, all at sub‐inhibitory concentrations (0.5 MIC). Green arrows indicate up‐regulation, red indicate down‐regulation, blue indicate unique to UA exposed cells, brown indicate unique in Nor exposed cells and yellow indicate proteins unique in cells exposed to UA–norfloxacin combination.
**Fig. S5.** Functional clustering and biological pathways using DAVID and PANTHER tools associated with the differentially expressed proteins identified in UA–norfloxacin treated MRSA‐2071 cells. Pie chart showing the protein classes (A), biological processes (B) and molecular functions (C).
**Fig. S6.** Protein–protein interaction network of differentially expressed proteins. The differentially expressed proteins in MRSA‐2071 cells in response to UA–norfloxacin combination were searched for their protein–protein interactions using the web resource STRING v.10.0 (http://www.string-db.org/). Protein–protein interactions are shown in evidence view and proteins are linked based on neighborhood, gene fusion, co‐occurrence, co‐expression, experimental evidences, existing databases and text mining. Network analysis was set at high stringency (STRING score ≥ 0.7).
**Fig. S7.** Time kill assay of different MRSA strains alone as well as in combination in three different clinical isolates namely MRSA‐1745, MRSA‐4627 and MRSA‐5944. These data represent mean ± SEM of three independent experiments.
**Fig. S8.** Efflux pump modulatory potential of UA in clinical isolates MRSA‐1745, MRSA‐4627 and MRSA‐5944 using EtBr as marker, determined through spectrofluorometric analysis. Reserpine, an efflux pump inhibitor, was used as positive control in the experiment.
**Fig. S9.** Prospecting membrane disruptive property of UA–norfloxacin combination against clinical isolates MRSA‐1745, MRSA‐4627 and MRSA‐5944 using PI through spectrofluorimetry. These data represent mean ± SEM of three independent experiments (**P ≤ 0.01, ***P ≤ 0.001, Dunnett's test).
**Fig. S10.** Membrane depolarization assay of UA–norfloxacin combination using diSC_3_‐5 against clinical isolates MRSA‐1745, MRSA‐4627 and MRSA‐5944 via spectrofluorimetry. These data represent mean ± SEM of three independent experiments (**P ≤ 0.01, ***P ≤ 0.001, Dunnett's test).
