# Supplementary material for: Usnic acid modifies MRSA drug resistance through down‐regulation of proteins involved in peptidoglycan and fatty acid biosynthesis
Source: FEBS Open Bio. 2019 Sep 30;9(12):2025–40. doi: 10.1002/2211-5463.12650 (PMC6886298; doi:10.1002/2211-5463.12650)

**Usnic acid modifies modifies MRSA drug resistance through down-regulation of proteins involved in peptidoglycan and fatty acid biosynthesis**

*Sneha Sinha^1^, Vivek Kumar Gupta^1*^, Parmanand Kumar^1^, Rajiv Kumar^2^, Robin Joshi^2^, Anirban Pal^1^, Mahendra P Darokar^1#^*

*^1^ Molecular Bioprospection Department, CSIR-Central Institute of Medicinal and Aromatic Plants, Lucknow-226015, India*

*^2^ Biotechnology Division, CSIR-Institute of Himalayan Bioresource Technology, Palampur-176061, Himachal Pradesh, India*

**Present address: Department of Biotechnology, Indian Institute of Technology, Roorkee-247667, India*

**#** **Address of Corresponding author**:

Mahendra P. Darokar, Molecular Bioprospection Department, CSIR-Central Institute of Medicinal and

Aromatic Plants (Council of Scientific and Industrial Research), P.O. CIMAP, Lucknow-226 015, India,

Tel: +91-522-2718532; Fax: +91-522-2342666

Email: mpdarokar@yahoo.com, mp.darokar@cimap.res.in

**Running title**: **Usnic acid synergises with norfloxacin against MRSA**

**Abbreviations**:

UA: Usnic Acid; MRSA: methicillin-resistant *S. aureus*; HA: Hospital-acquired; CA: Community-acquired; VRSA: Vancomycin-resistant *S. aureus*; MDR: Multidrug resistance; CFU: Colony forming units; ROS: reactive oxygen species; MALDI-TOF: Matrix-assisted laser desorption ionization time-of-flight; NO: Nitric oxide; DAPI: 4’6-diamidino-2-phenylindole; OD: Optical density; SDS-PAGE: Sodium dodecyl sulphate polyacrylamide gel electrophoresis; NBT: nitro blue tetrazolium; BCIP: 5-bromo-4-chloro-3-indolyl phosphate, ED_25_: 25% of effective dose; ED_50_: 50% of effective dose; ESBL: Extended-spectrum beta-lactamase

**Table S1**: Source of clinical isolates used in the present study

*MTCC: Microbial Type Culture Collection

| ***S. aureus* strains** | **Source** | |
| --- | --- | --- |
| SA-96 | MTCC | CSIR-IMTECH, Chandigarh, India |
| SA-2071 | Sputum | SGPGI, Lucknow, India |
| SA-1745 | Sputum |
| SA-5944 | Blood |
| SA-4627 | Pus |  |
| SA-3151 | Sputum |
| SA-4423 | Pus |
| SA-4620 | Pus |
| SA-10760 | Blood |

**Table S2**: Antibiotic sensitivity/resistance profiling of clinical isolates of *S. aureus*

| **ANTIBIOTICS** | **MIC (mg/l)** | | | | | | | | |
| --- | --- | --- | --- | --- | --- | --- | --- | --- | --- |
|  | **SA-96** | **SA-2071** | **SA-1745** | **SA-5944** | **SA-4627** | **SA-3151** | **SA-4423** | **SA-4620** | **SA-10760** |
| Vancomycin | 1.56 | 3.125 | 6.125 | 1.56 | 1.56 | 1.56 | 1.56 | 1.56 | 3.125 |
| Oxacillin | 0.78 | 1000 | 1000 | 1000 | 500 | 500 | 500 | 1000 | 250 |
| Tetracycline | 0.78 | 25 | 50 | 12.5 | 50 | 25 | 25 | 25 | 50 |
| Norfloxacin | 0.78 | 500 | 500 | 500 | 250 | 500 | 250 | 250 | 500 |
| Nalidixic acid | 1.56 | >100 | 500 | 500 | 250 | 1000 | 2000 | 2000 | >1000 |
| Ampicillin | 0.195 | >100 | 62.5 | >1000 | >100 | 500 | 500 | 1000 | 500 |
| Neomycin | 1.56 | 0.78 | 0.39 | 31.25 | >100 | 250 | 250 | 250 | 250 |
| Ofloxacin | 12.5 | >100 | 500 | 500 | 1000 | 500 | 500 | 250 | 1000 |
| Penicillin | 0.195 | 1000 | 1000 | 1000 | >1000 | 500 | 500 | 1000 | 500 |
| Kanamycin | 3.125 | 500 | 125 | 1000 | 500 | 125 | 250 | 250 | 250 |
| Streptomycin | 12.5 | 500 | 500 | 1.56 | 125 | 125 | 250 | 250 | 250 |
| Ciprofloxacin | 0.78 | 500 | 500 | 125 | 125 | 250 | 125 | 250 | 125 |
| Rifampicin | 0.78 | >100 | >100 | >100 | 6.125 | 1.56 | 3.125 | 1.56 | 1.56 |
| Cefoxitin | 1.56 | >100 | >100 | >100 | >100 | 500 | 250 | 250 | 500 |
| Erythromycin | 1.56 | >100 | >100 | >100 | >100 | 250 | 250 | 125 | 250 |
| Linezolid | 0.07 | 0.62 | 1.25 | 0.62 | 1.25 | 1.56 | 1.56 | 3.125 | 1.56 |
| Daptomycin | 0.625 | 0.625 | 0.625 | 1.25 | 1.25 | 0.625 | 1.25 | 2.5 | 1.25 |
| Tigecycline | 1.25 | 1.25 | 2.5 | 1.25 | 10 | 1.25 | 1.25 | 0.625 | 1.25 |
| Teicoplanin | 0.78 | 3.125 | 3.125 | 3.125 | 1.56 | 1.56 | 1.56 | 0.78 | 1.56 |

**Table S3**: Mutation frequency of *S. aureus* (MTCC-96) with UA and norfloxacin

| **Agent** | **MIC** | **2MIC** | **4MIC** | **8MIC** | **16MIC** |
| --- | --- | --- | --- | --- | --- |
| UA | 4.22 x 10^-10^ | 2.87 x 10^-10^ | 1.5 x 10^-10^ | 0.61 x 10^-10^ | < 10^-10^ |
| Norfloxacin | 3.4 x 10^-10^ | 1.92 x 10^-10^ | 0.48 x 10^-10^ | < 10^-10^ | < 10^-10^ |
| UA + Norfloxacin | 2.53 x 10^-10^ | 0.34 x 10^-10^ | < 10^-10^ | < 10^-10^ | < 10^-10^ |

**Table S4**: List of primers used in the present study

| **Gene name** | **Primer sequence (5'-3')** |
| --- | --- |
| mecA-F | AAAATCGATGGTAAAGGTTGGC |
| mecA-R | AGTTCTGGAGTACCGGATTTGC |
| eno-F | GGTGCTACAACGTTCAAAGAATCA |
| eno-R | TTCAAATTTAGGAGCGAAACCACC |
| qoxA-F | TCTTTCACCCATTTGTCGTAGTCT |
| qoxA-R | GAAGCATCTCAAACTGGTACGTTC |
| grpE-F | TTGGATCAAATGCTTCACCTTCAG |
| grpE-R | CCAGCAATAGACAATATAGAACGTGC |
| scdA-F | CATTGCAACGGGAGAATTGTTAGT |
| scdA-R | TCTCTGATAAACAAGTCGCCAAGT |
| tkt-F | TGGGAAGTTGCCTTAGAATCTGAA |
| tkt-R | ACGCCTTCTTCAACTACATCTTCT |
| sodA-F | GGTTCAGGTTGGGCTTGGT |
| sodA-R | TCTTGGTTTGGTGTAGTCACAATTTC |
| ahpC-F | CGTAAAAACCCTGGCGAAGTAT |
| ahpC-R | TGCAATGTTTTAGCGCCTTCT |
| katA-F | AATGTTTGCCCGTTTCTCTACTG |
| katA-R | TGTCACGCTCCGCATCAG |
| tpx-F | CACCGTGACTTATCATTTGGTGAA |
| tpx-R | GCACGAGCTAATAAGCGAAGTTC |
| ftnA-F | AATCGTACGAAGGATTTGCAAAC |
| ftnA-R | CTTTTGTCCATGGAAACGTTCTT |
| perR-F | GCGACAAGCAGGCGTAAGA |
| perR-R | GCTGTTGGATGAGTATGTGAAGAAA |
| tuf-F | TGTTACAGGTGTTGAAATGTTCCG |
| tuf-R | TTGAACCAGGAGCAGCTAATACTT |
| groEL-F | CTGTTTCAATGTCACCTTCAGCTT |
| groEL-R | GCAGCAGTTGAAGAAGGTATTGTT |
| rplQ-F | TAACCACCTTGACGTTCTGTGTAA |
| rplQ-R | TTCTCGTCGTAATGCAGCTAAAAC |
| fabI-F | CAAAAGGTGTGGGTGGTTTCAATA |
| fabI-R | AAGCCGCAGTTTTACCTACTTCTA |
| fabH-F | AGTAACAGGTGCATCAAGAGGAAT |
| fabH-R | TCGCAAAACTGTCAACACCTTTAG |
| ftsK-F | CAAAAGGCACGTCAAAATGAACAG |
| ftsK-R | GACTTGGTTGACTCTGGCTTTTAC |
| femX-F | CATGTTCACGTGCATACTTCATCA |
| femX-R | GCACTATTAATGTTTGCTGGCTCA |
| tig-F | GCTCTTCGAAACCAGGTATGAATG |
| tig-R | TTTAGTGGTTCAGTTGACGGAGAA |
| murA-F | ACGTTCAATGTCTGCACCTAATTG |
| murA-R | TAGTTGCTGATGGTAAAACAAGCG |
| murF-F | TCTTCAACCCATATAATAGGCCCG |
| murF-R | AAGGTGAAAATGTTGACGGTCATC |
| norA-F | TCGTCTTAGCGTTCGGTTTA |
| norA-R | TCCAGTAACCATCGGCAATA |
| norB-F | ATGGAAAAGCCGTCAAGAGA |
| norB-R | AACCAATGATTGTGCAAATAGC |
| norC-F | ATGAATGAAACGTATCGCGG |
| norC-R | GTCTGCACCAAAACTTTGTTGTAAA |
| mepA-F | TGCTGCTGCTCTGTTCTTTA |
| mepA-R | GCGAAGTTTCCATAATGTGC |
| mdeA-F | GTTTATGCGATTCGAATGGTTGGT |
| mdeA-R | AATTAATGCAGCTGTTCCGATAGA |
| GAPDH-F | ACTTACGAGCAGATCAAAGC |
| GAPDH-R | AGTTTCACGAAGTTGTCGTT |

**Figure S1:**


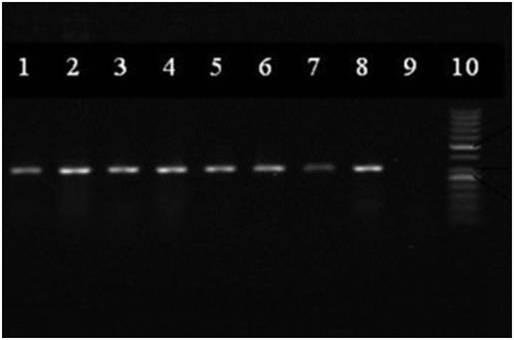


**Figure S2:**


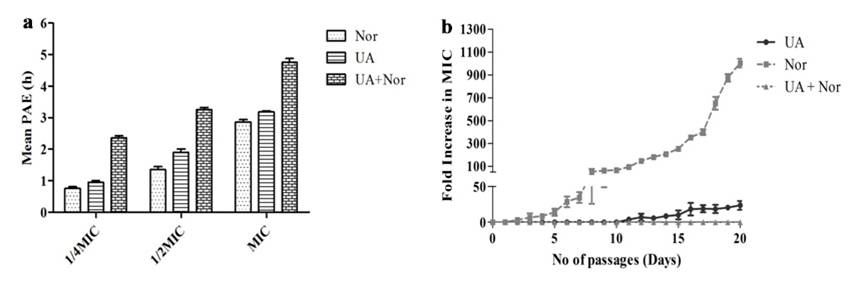


**Figure S3:**

**
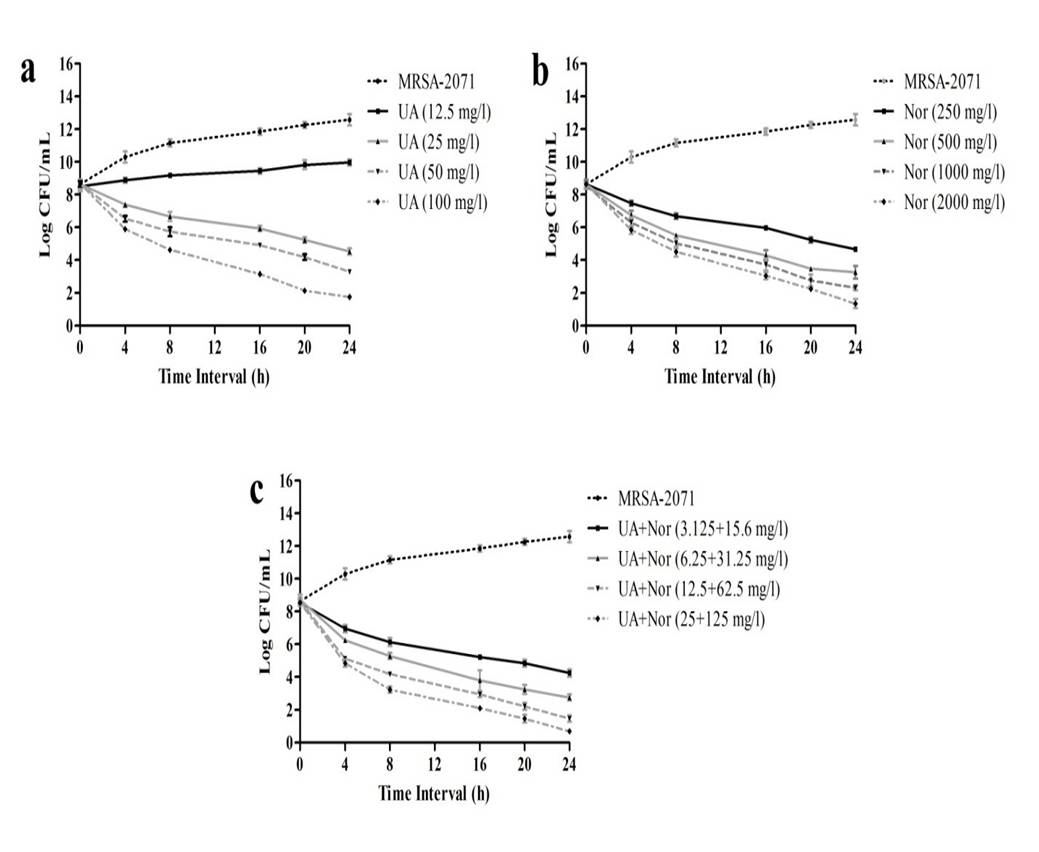
**

**Figure S4(a):**

**
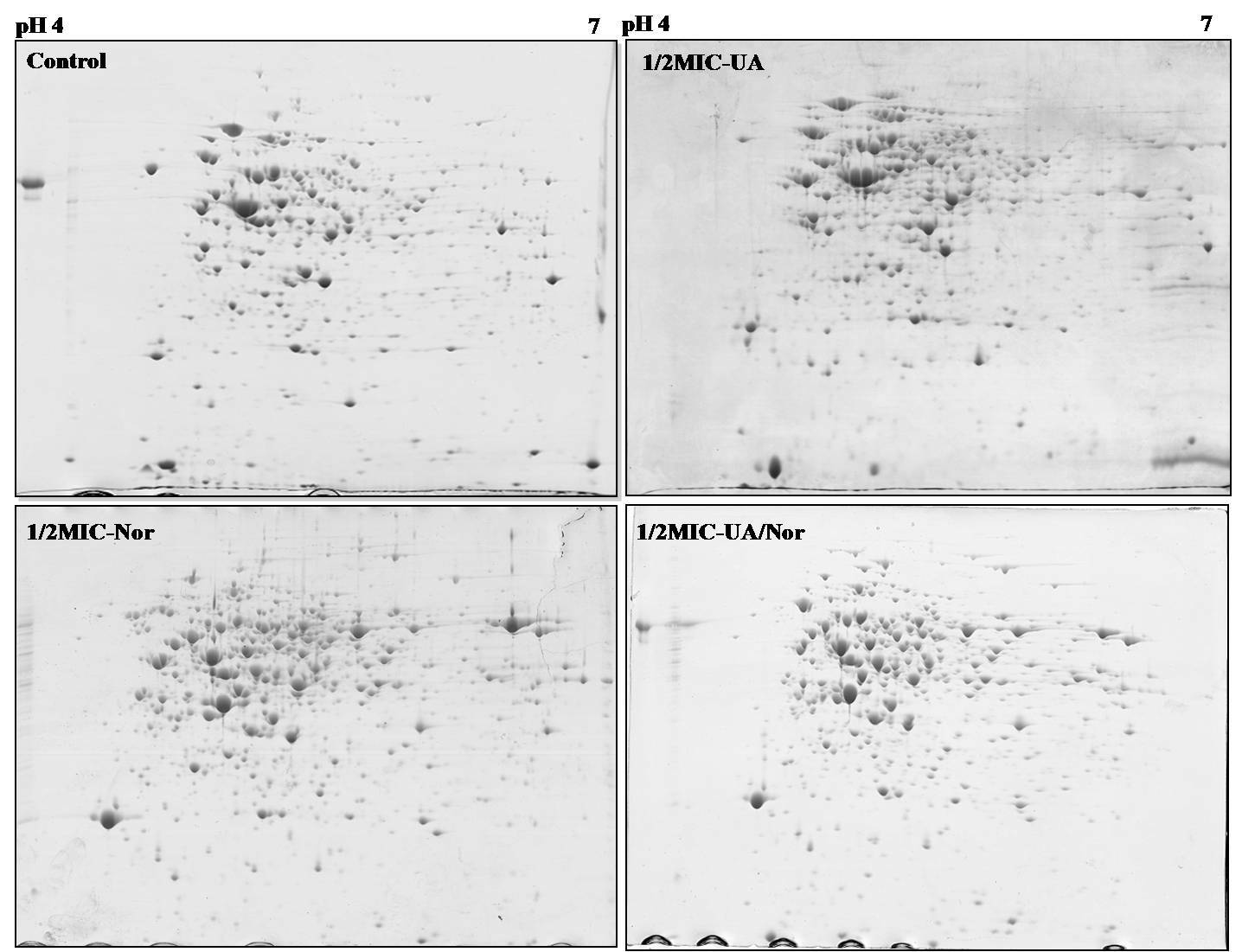
**

**Figure S4(b):**

**
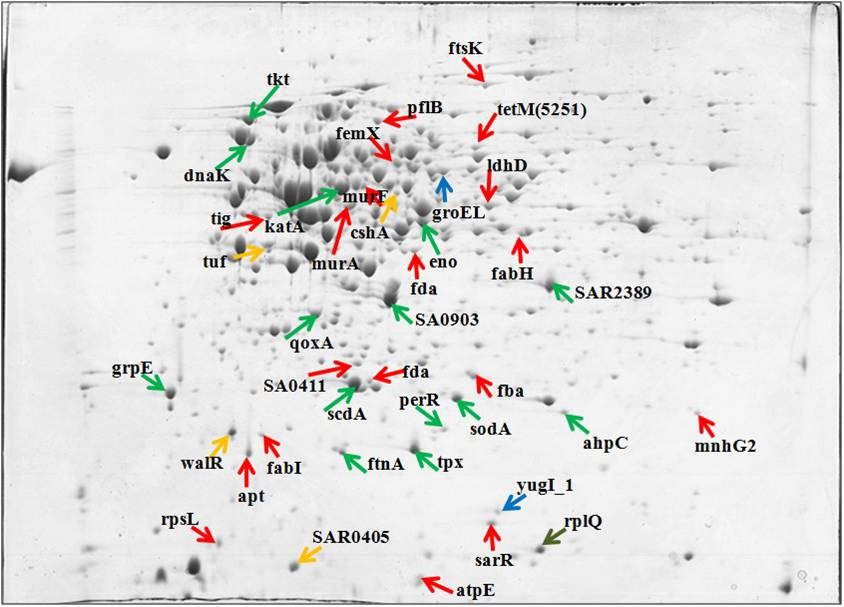
**

**Figure S5:**


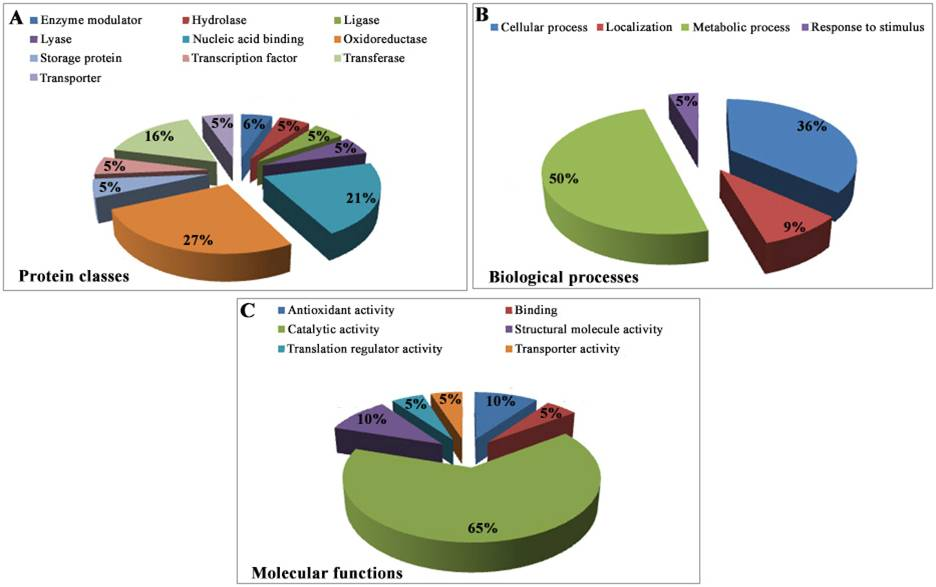


**Figure S6:**


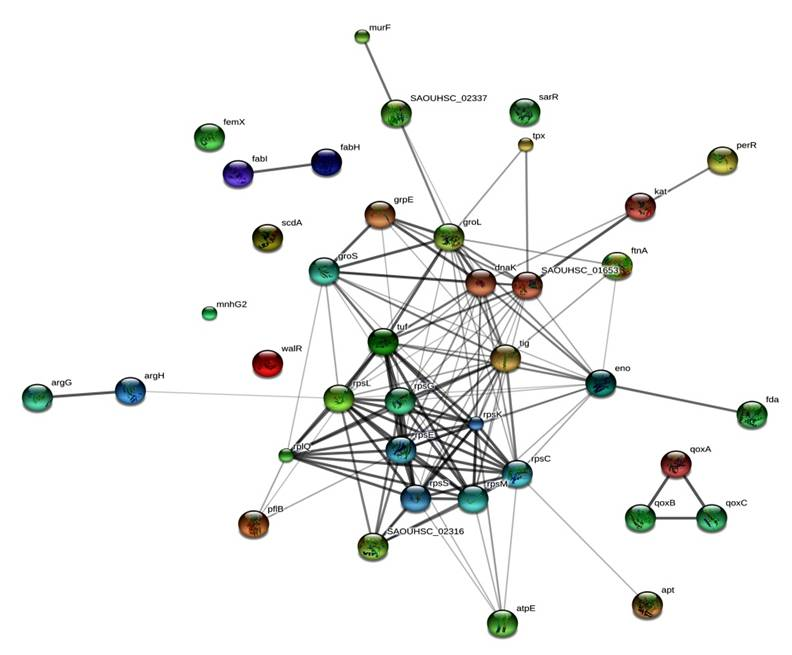


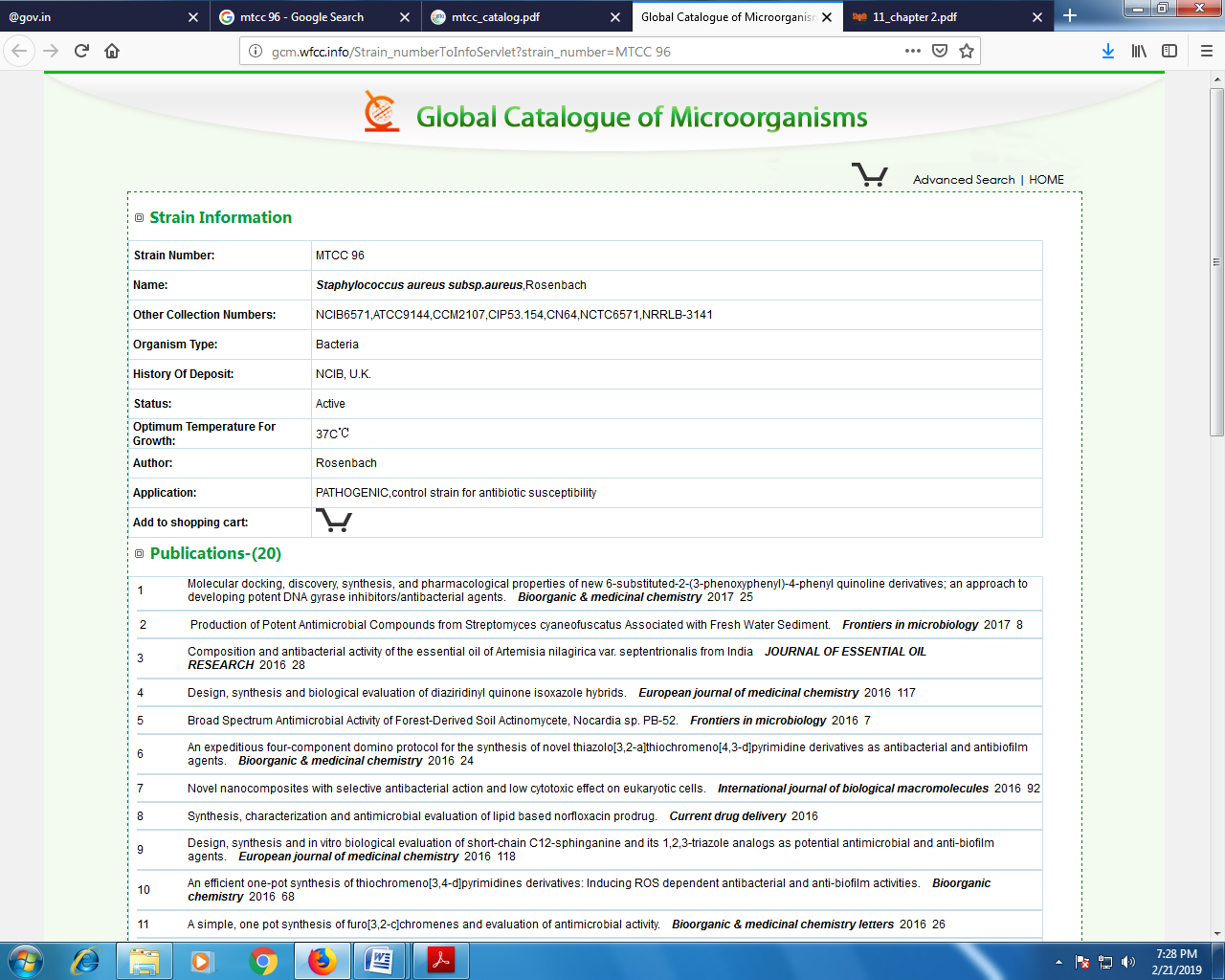
**For reference only**

**Figure S7:**

Time Kill Curves of different MRSA strains alone as well as in combination showed similar dose-dependent killing.

**
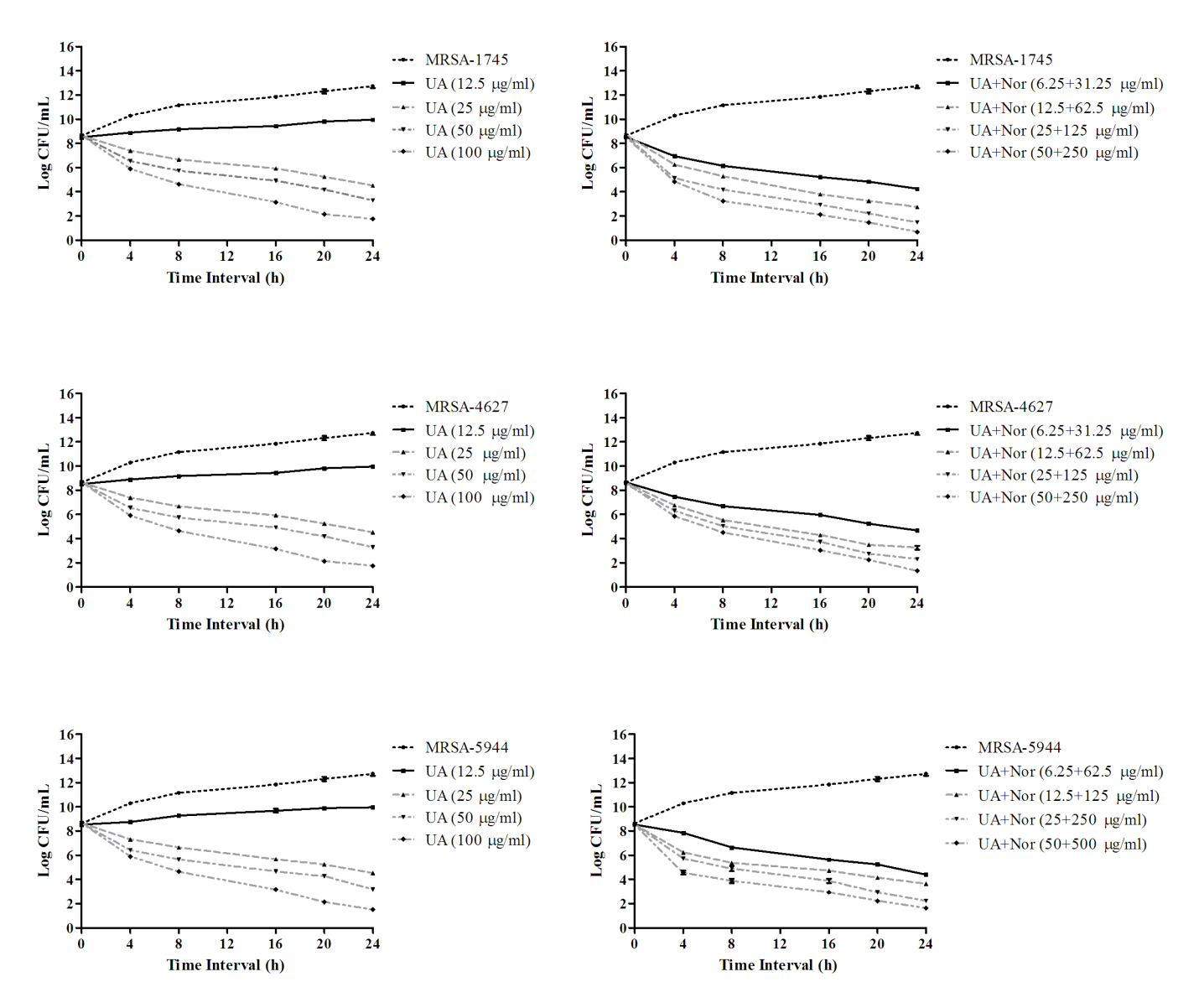
**

**Figure S8:**

Efflux pump inhibitory potential of UA was also investigated in different MRSA isolates as well.


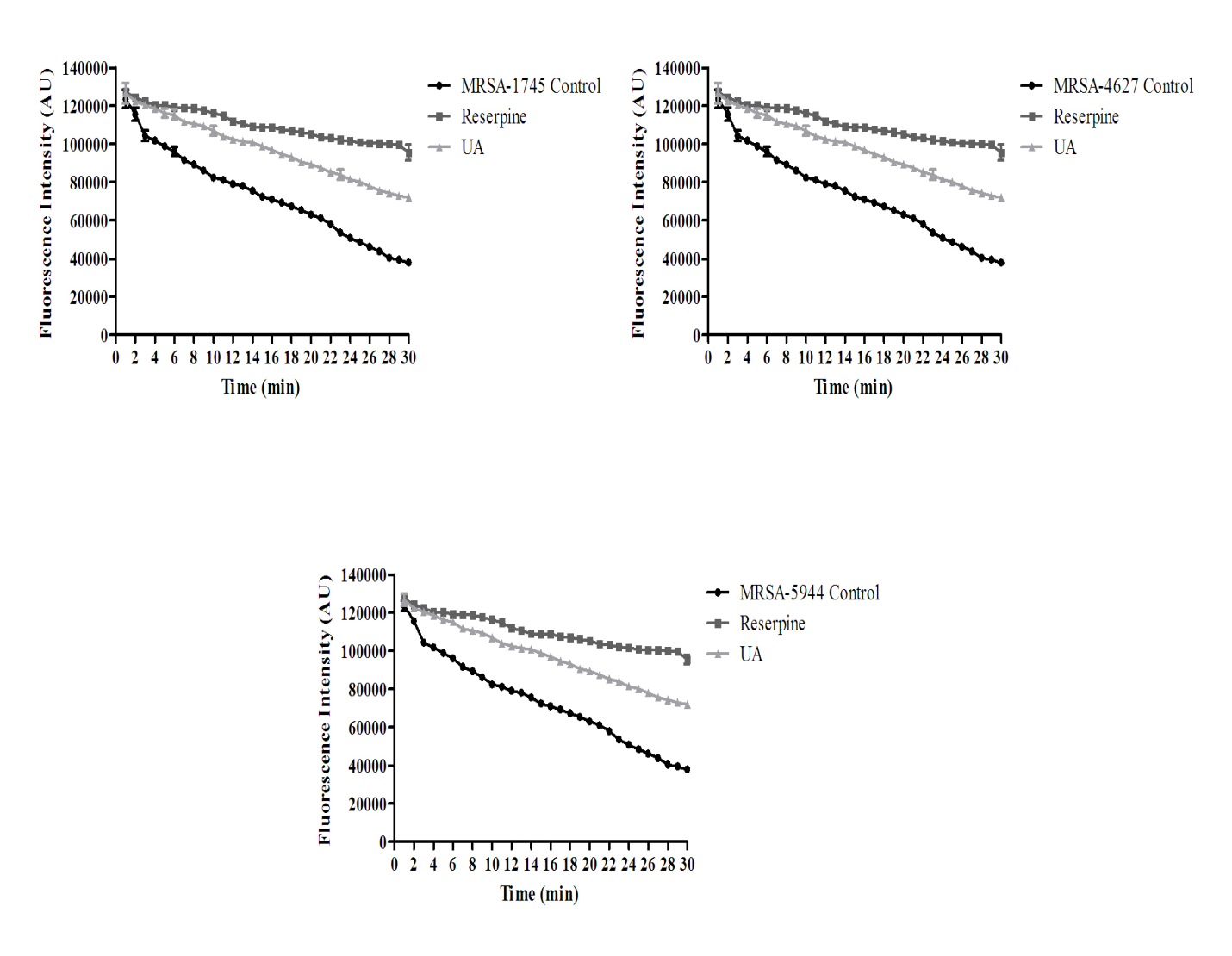


**Figure S9:**

Membrane permeabilization assay using Propidium Iodide (PI) was also investigated in the presence of UA alone as well as in combination with norfloxacin in other MRSA isolates which also displayed that UA indeed alteration in membrane permeability causing increased PI fluorescence in cells exposed to UA in dose-dependent manner.

**
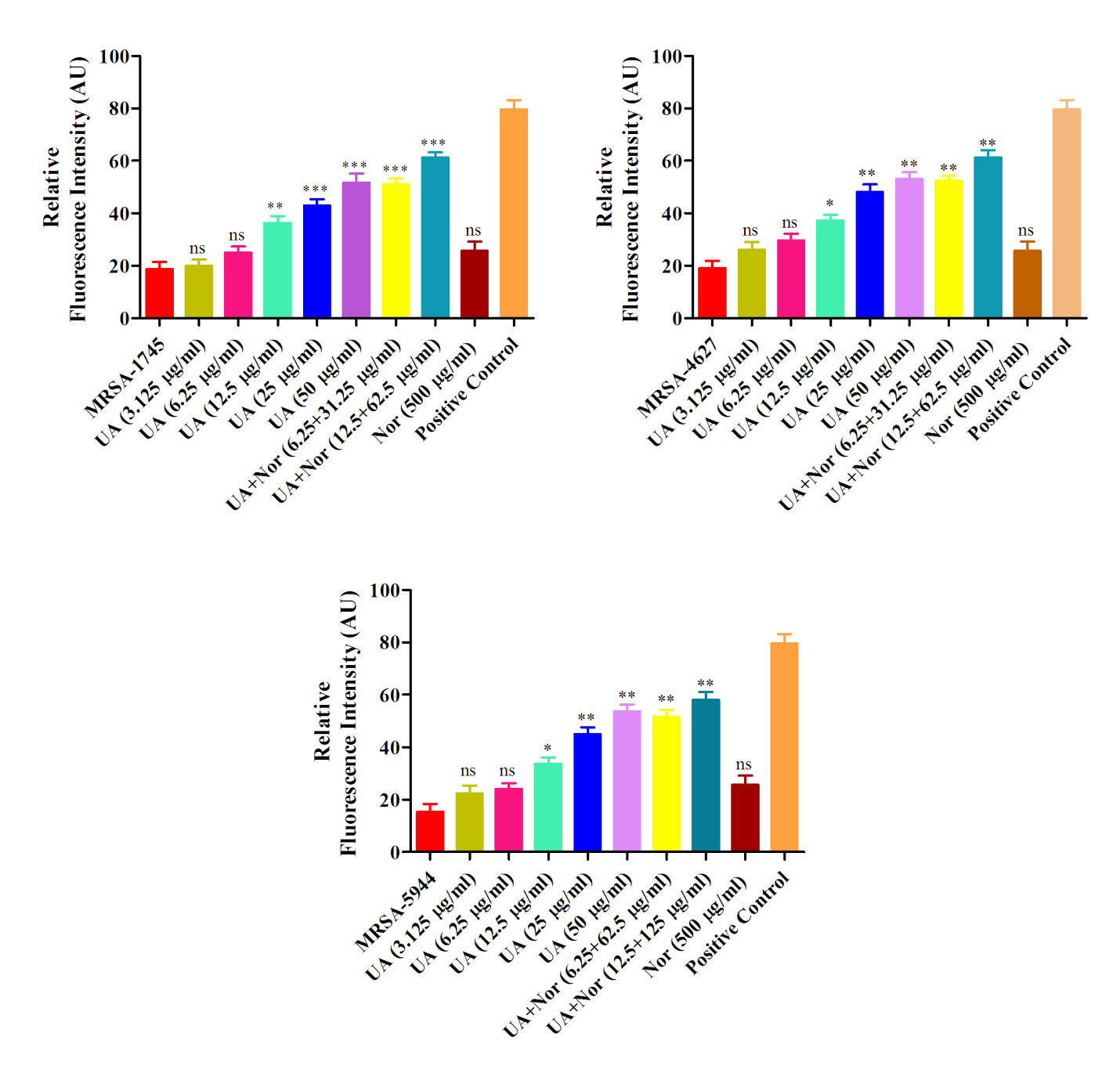
**

**Figure S10:**

Membrane depolarization assay using diSC_3_-5 was also studied in other MRSA isolates and increased fluorescence highlights the disruption in the membrane potential in these as well, in accordance with the MRSA-2071 strain reported in the main text.


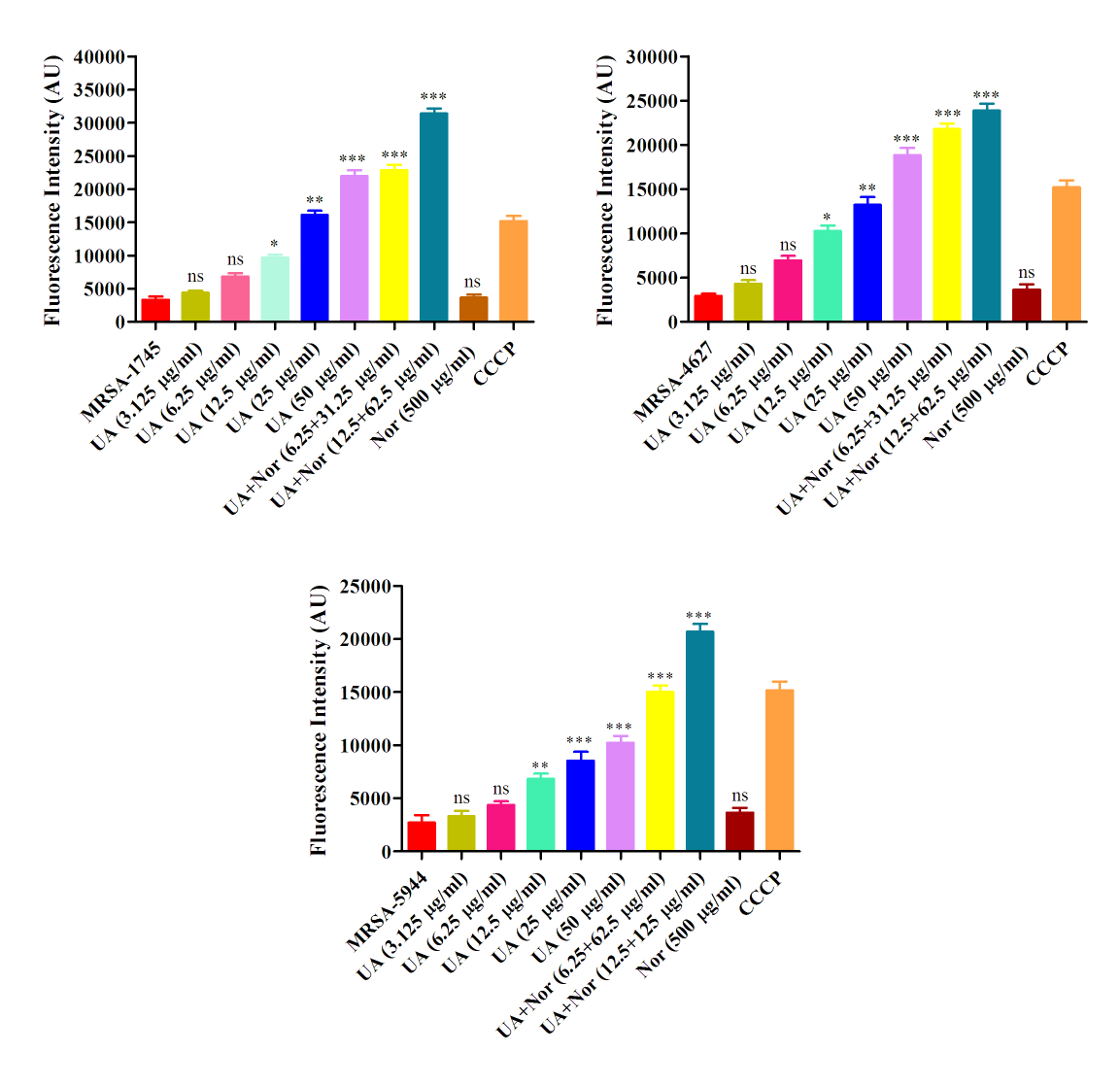

Supplement: Supplementary file 1 — Table S1. Source of clinical isolates used in the present study. Table S2. Antibiotic sensitivity/resistance profiling of clinical isolates of S. aureus. Table S3. Mutation frequency of S. aureus (MTCC‐96) with UA and norfloxacin. Table S4. List of primers used in the present study. Fig. S1. PCR amplification of mecA gene from the clinical isolates of S. aureus. (Lane 1: SA‐2071, Lane 2: SA‐1745, Lane 3: SA‐5944, Lane 4: SA‐4627, Lane 5: SA‐3151, Lane 6: SA‐4423, Lane 7: SA‐10760, Lane 8: SA‐4620, Lane 9: MTCC SA‐96, Lane 10: Ladder.) Fig. S2. (a) PAE of norfloxacin alone as well as in combination with UA against clinical isolate MRSA‐2071. (b) Propensity of development of resistance of S. aureus against UA and norfloxacin combination compared to norfloxacin alone. Fig. S3. Time kill kinetics of MRSA‐2071 at different concentrations of UA and norfloxacin alone as well as in combination. These data represent mean ± SEM of three independent experiments. Fig. S4. (a) Protein expression profiles of soluble proteins from MRSA‐2071 cells exposed to sub‐lethal concentrations of UA and norfloxacin alone as well as in combination. Proteins were resolved on 17 cm pH 4–7 IPG strips and 12.5% 20 cm SDS/PAGE gels. (½ MIC UA‐ 12.5 mg·L−1; ½ MIC Nor‐ 250 mg·L−1; ½ MIC UA/Nor‐ 3.125 + 15.6 mg·L−1). (b) Temporal proteome changes of MRSA‐2071 under UA–norfloxacin treatment identified using classical two‐dimensional electrophoresis. Proteins were separated in first dimension isoelectric focussing on 17 cm IPG strips of pH 4–7 range followed by second dimension separation on 12.5% SDS/PAGE. Multivariate analysis was performed to identify the differentially expressing protein spots in response to UA–norfloxacin treatment compared to untreated control and UA and norfloxacin alone, all at sub‐inhibitory concentrations (0.5 MIC). Green arrows indicate up‐regulation, red indicate down‐regulation, blue indicate unique to UA exposed cells, brown indicate unique in Nor exposed cells and yellow [file FEB4-9-2025-s001.doc]
